# Research Progress of Electropulsing-Assisted Machining Technology for Difficult-to-Machine Metal Materials

**DOI:** 10.3390/ma19173792

**Published:** 2026-09-06

**Authors:** Jingwei Sun, Shengjun Xia, Chunfa Huang, Qiulin Li

**Affiliations:** 1 Institute of Materials Research, Shenzhen International Graduate School, Tsinghua University, Shenzhen 518055, China; 2 School of Materials Science and Engineering, Tsinghua University, Beijing 100084, China

**Keywords:** electropulsing, Joule heating effect, athermal effects, difficult-to-machine metallic materials, machining technology

## Abstract

Difficult-to-machine metallic materials, distinguished by their high strength, hardness, and corrosion resistance, are indispensable in critical fields such as aerospace and defense. However, these very attributes also pose substantial challenges to conventional machining technologies. As an advanced external-field machining technology, electropulsing-assisted machining provides unique advantages in enhancing plastic deformability, refining microstructures, improving machining precision, and boosting efficiency. This paper provides a comprehensive review of the fundamental mechanisms governing the interaction between electropulsing and metallic materials, synthesizes the latest advances in electropulsing-assisted machining methods, and highlights unresolved challenges alongside promising future directions. By bridging the gap between material science and advanced manufacturing, this review aims to stimulate innovation and accelerate the adoption of electropulsing-assisted machining in the processing of next-generation high-performance metallic materials.

## 1. Introduction

Difficult-to-machine metal materials possess high strength [1], high hardness [2], and remarkable corrosion resistance [3]. Extensively employed in aerospace, defense industries, as well as other high-end manufacturing sectors [4,5]. Conventional processing procedures, such as turning and rolling, always produce severe work-hardening, giving rise to significant tool wear [6,7,8], suboptimal machining quality [9,10,11], low machining efficiency [12], and exorbitant machining costs [13,14]. Such drawbacks have restricted the wide application of these materials in the above-mentioned fields. Therefore, it is necessary to conduct research on new processing technologies for difficult-to-machine metal materials [15,16,17].

In recent years, electropulsing-assisted machining technology, as an emerging technology, has begun to be applied in the machining of difficult-to-machine metal materials [18]. This technology employs pulsed electric current on materials during processing, capitalizing on the high temperature, high pressure, high-speed impact, and electrochemical interaction induced by the pulsed current to augment the plastic deformation capacity of materials, leading to local softening and enabling fast processing. This technology not only remarkably boosts machining efficiency and mitigates tool wear but also efficaciously enhances machined surface quality. It thus paves a novel path for the high-quality machining of difficult-to-machine metal materials and facilitates their broader application. Currently, both electropulsing-assisted machining technology and the underlying mechanism of the impact of electropulsing have been the subject of extensive research. Tang et al. [19,20,21] investigated a multiplicity of electropulsing-assisted machining technologies and devised a comprehensive machining system, one which has spurred the development of electropulsing-assisted machining technology. Lv et al. [22,23,24] analyzed the influence of electropulsing on the structure and properties of materials from the perspectives of thermal and athermal effects.

This paper mainly reviews the research progress of electropulsing-assisted machining technology. Initially, the effect of electropulsing on materials and its mechanism is analyzed. Subsequently, the burgeoning electropulsing-assisted machining technologies for difficult-to-machine metal materials are introduced and the surface and mechanical properties of materials after machining are collated. Finally, the extant challenges and prospective development directions regarding electropulsing-assisted machining technology are delineated, with the aim of furnishing certain references for the advancement of this field.

## 2. Effect of Electropulsing on Materials and Its Mechanism

### 2.1. Effect of Electropulsing on Materials

Electropulsing is capable of elevating the temperature of materials, facilitating the directional migration of electrons and promoting the diffusion of atoms and vacancies. Consequently, when electropulsing is applied to materials, it will exert a significant influence, including on slip system activation, texture evolution, grain refinement, and phase transformation. These aspects will be elaborated in detail in this section.

#### 2.1.1. Induced Slip System Activation

**Figure 1:**
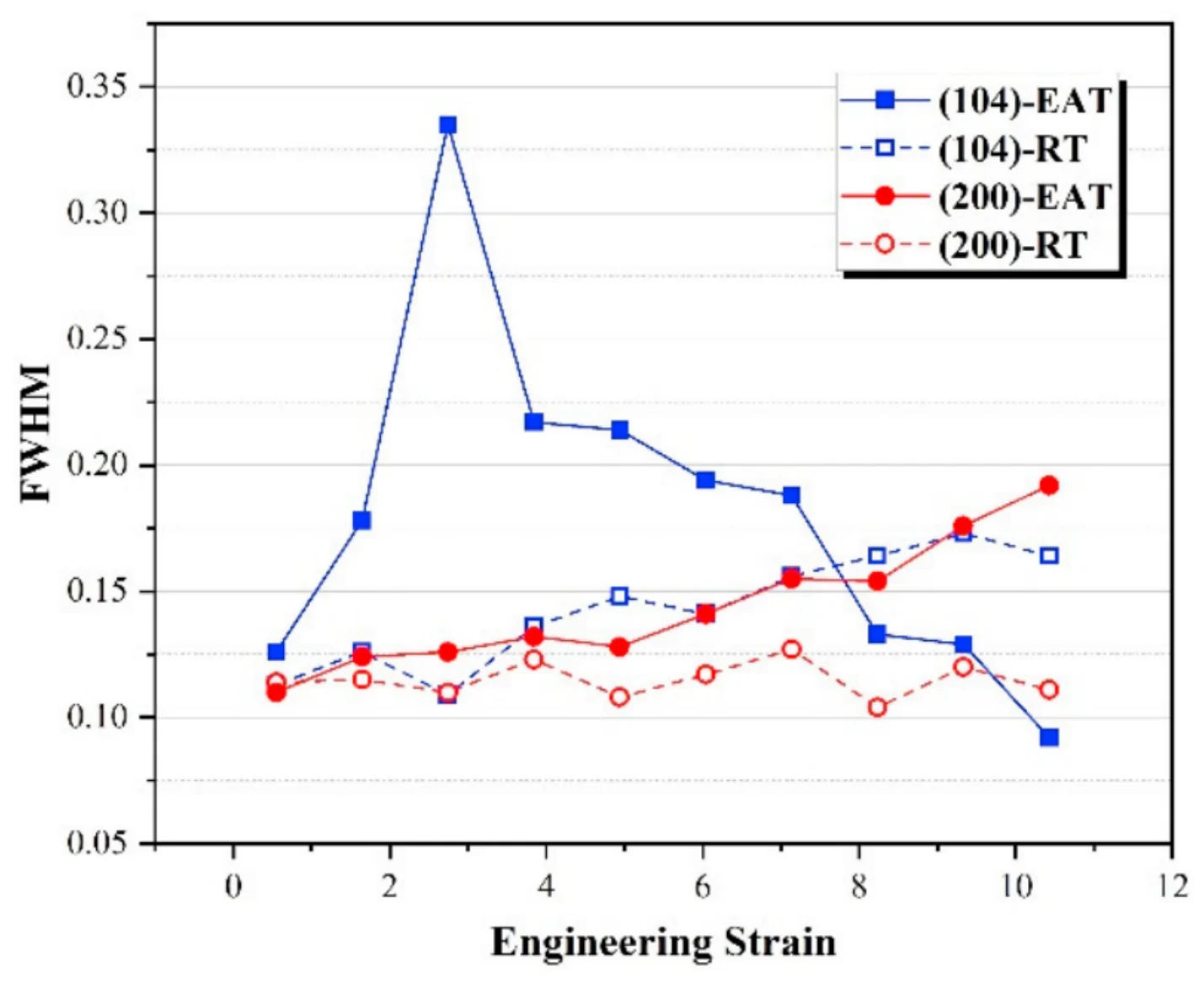
Variation of half peak width of X-ray diffraction peaks with different crystal planes in TC4 alloy [29].

During the plastic deformation of difficult-to-machine materials, moving dislocations can be pinned by defective particles like second-phase particles and impurities. This results in the blockage of their motion and makes the plastic deformation of materials more difficult. The application of electropulsing to materials can trigger the initiation of new slip systems and facilitate dislocation motion [25,26], thereby enhancing the plastic deformation of materials [27,28]. Li et al. [29] analyzed the variation in X-ray diffraction peaks of full width at half maximum (FWHM) for different crystal planes in TC4 alloy after electropulsing-assisted stretching (EAT), as depicted in Figure 1. Compared with the curve corresponding to the situation where no electropulsing was applied (RT), the half width of the peak for the (200) crystal plane gradually increased with the increase in strain after electropulsing was applied. This indicates that electropulsing activates the prismatic slip system of a hexagonal close-packed structure, resulting in an increase in the number of dislocations within this slip system. Meanwhile, the half-peak width of the (104) crystal plane first increases and then decreases with the increase in strain. This indicates that the pyramidal slip system of a hexagonal close-packed structure is activated in the early stage of strain, generating a large number of dislocations, and then the annihilation of dislocations causes the number of dislocations in this slip system to decrease as the strain increases.

#### 2.1.2. Promote Texture Evolution

During the plastic deformation of materials, electropulsing can eliminate textures formed during processing and create new textures by promoting recrystallisation and other means [30,31,32,33,34]. Materials with different crystal structures exhibit different forms of texture changes [35]. Kumar et al. [36] observed the austenitic matrix of Fe-18.17%Mn-10.51%Al-1.04%C-5.88%Ni steel (which has an FCC crystal structure) after electropulsing treatment. They found that the deformation textures of Goss {110} <001> and S-type disappeared and that new textures such as (012) <021>, (021) <102>, and (210) <210> were generated. Zhang et al. [37] investigated the microstructure of M50 steel (with a BCC crystal structure) in the electropulsing-assisted forming process. They discovered that as the pulse current density increased, the strength of Cube {100} <001> and Goss {110} <001> textures gradually decreased and eventually disappeared, while the strength of newly generated α{110}//RD and *θ*{100}//TD textures gradually increased. Jiang et al. [38] examined the texture changes in AZ91 magnesium alloy (with an HCP crystal structure) after electropulsing treatment. They found that when the frequency of the pulse current increased to 133 Hz, the strength of {0001}<10$\bar{1}$0> texture increased significantly, whereas the strength of other textures decreased significantly.

**Figure 2:**
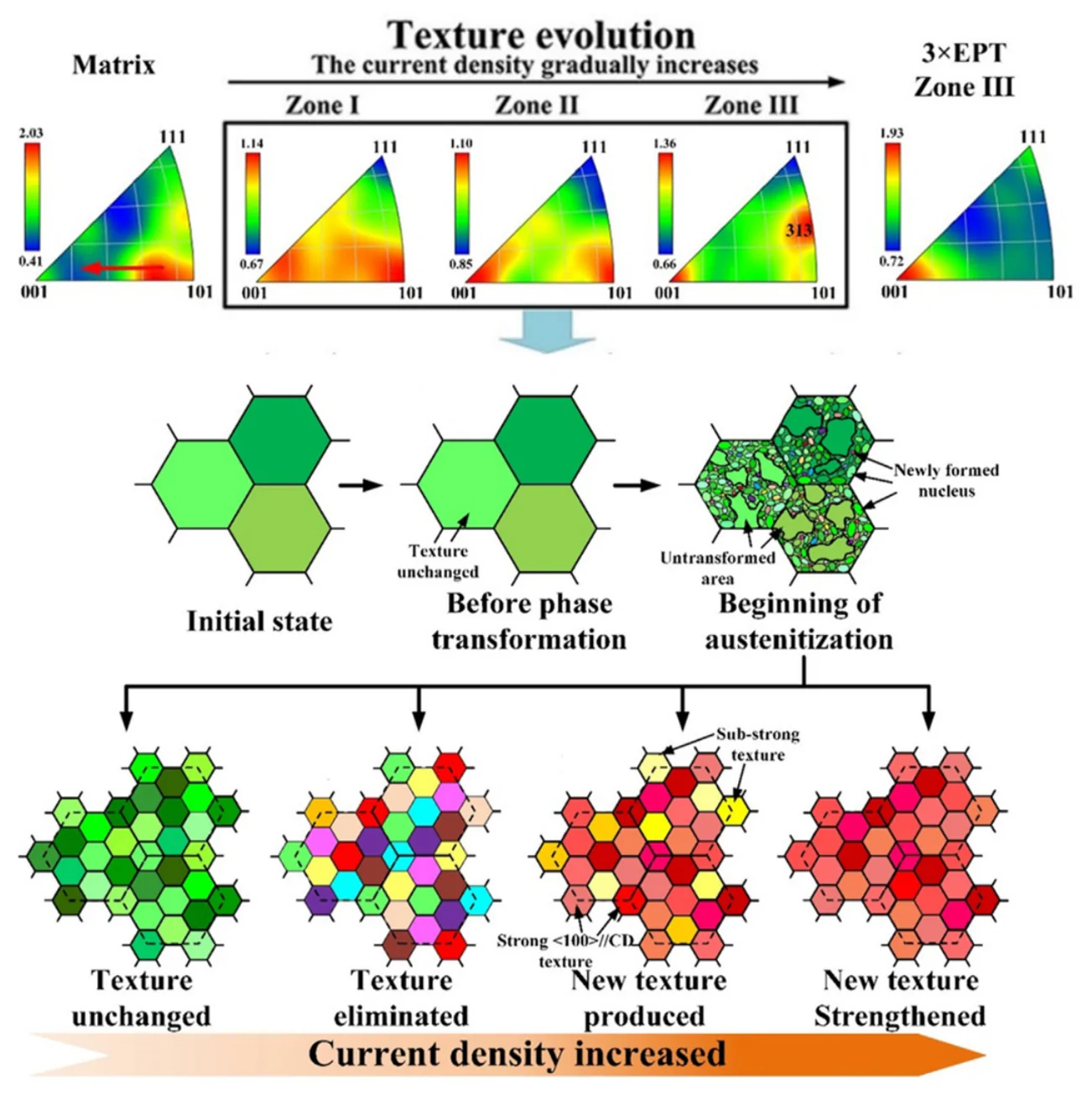
Variation of texture with pulse current intensity in 35CrMo steel [34].

In addition, electropulsing can modulate the direction of texture such that it aligns along the direction of lowest resistance, which coincides with the direction of current flow [39,40]. Wu et al. [34] applied electropulsing to hot rolled and annealed 35CrMo steel and discovered that when current density was low (336 A/mm^2^), the electropulsing could eliminate the hot rolled and annealed texture by inducing phase transformation. However, when the current density was high (1680 A/mm^2^), the grain resistance was low in the <100>//CD direction, so the current flowed along this direction, ultimately forming a new texture inside material along <100>//CD direction, as illustrated in Figure 2.

#### 2.1.3. Promote Grain Refinement

Electropulsing can facilitate grain refinement by promoting the recrystallisation process [41,42,43,44,45]. The mechanism by which electropulsing promotes the recrystallisation process will be described in Section 2.2.5. In addition, and on the one hand, electropulsing can promote the formation of subgrain boundaries and twinning boundaries to divide the original grains [32], while, on the other hand, electropulsing can reduce dislocation density inside materials, thereby reducing the driving growth force of recrystallized grains, thus inhibiting the growth of grains [22]. Duan et al. [46] discovered that the original average grain size of 25CrNi2MoV steel was approximately 12.7 μm, with most grains distributed within the range of 5–15 μm. Ultrasonic rolling treatment promoted the recrystallisation of material, refining the grains to 81.3 nm. Meanwhile, applying an electropulsing (with a frequency of 700 Hz and a peak current density of 2.264 A/mm^2^) could further promote the recrystallisation process and inhibit the growth of recrystallized grains, so that the grains were further refined to 15.3 nm and their grain size distribution approached a normal distribution.

#### 2.1.4. Regulate Phase Composition

Electropulsing can remarkably facilitate phase transition and modulate phase composition, primarily for the following reasons: (1) Electropulsing can cause a sharp temperature increase at defects, which triggers the nucleation of new phases. (2) Electropulsing can effectively enhance atomic diffusion and lower the activation energy of diffusion [47], thereby accelerating the nucleation process of new phases [48,49]. (3) Electropulsing can accelerate the recrystallisation process [50], which effectively induces the formation and growth of new phases. (4) The mismatch between new phases and parent phases at grain and subgranular boundaries is low, so the strain energy of nucleation is lower. This makes it easier for new phases to nucleate at grain and subgrain boundaries. Electropulsing accelerates the formation of grain and subgranular boundaries by promoting dislocation migration, which further promotes the nucleation of new phases [51]. (5) Electropulsing significantly reduces the activation energy for nucleation and growth of new phases [52], enabling the phase transition to be initiated at a higher nucleation rate and a lower temperature. (6) Dislocation annihilation is regarded as the driving force of phase transition [53]. Electropulsing can effectively promote dislocation evolution, thus facilitating the phase transition process. (7) Electropulsing can promote grain boundary migration, which increases the growth rate of new phases and thus promotes the phase transition process.

**Figure 3:**
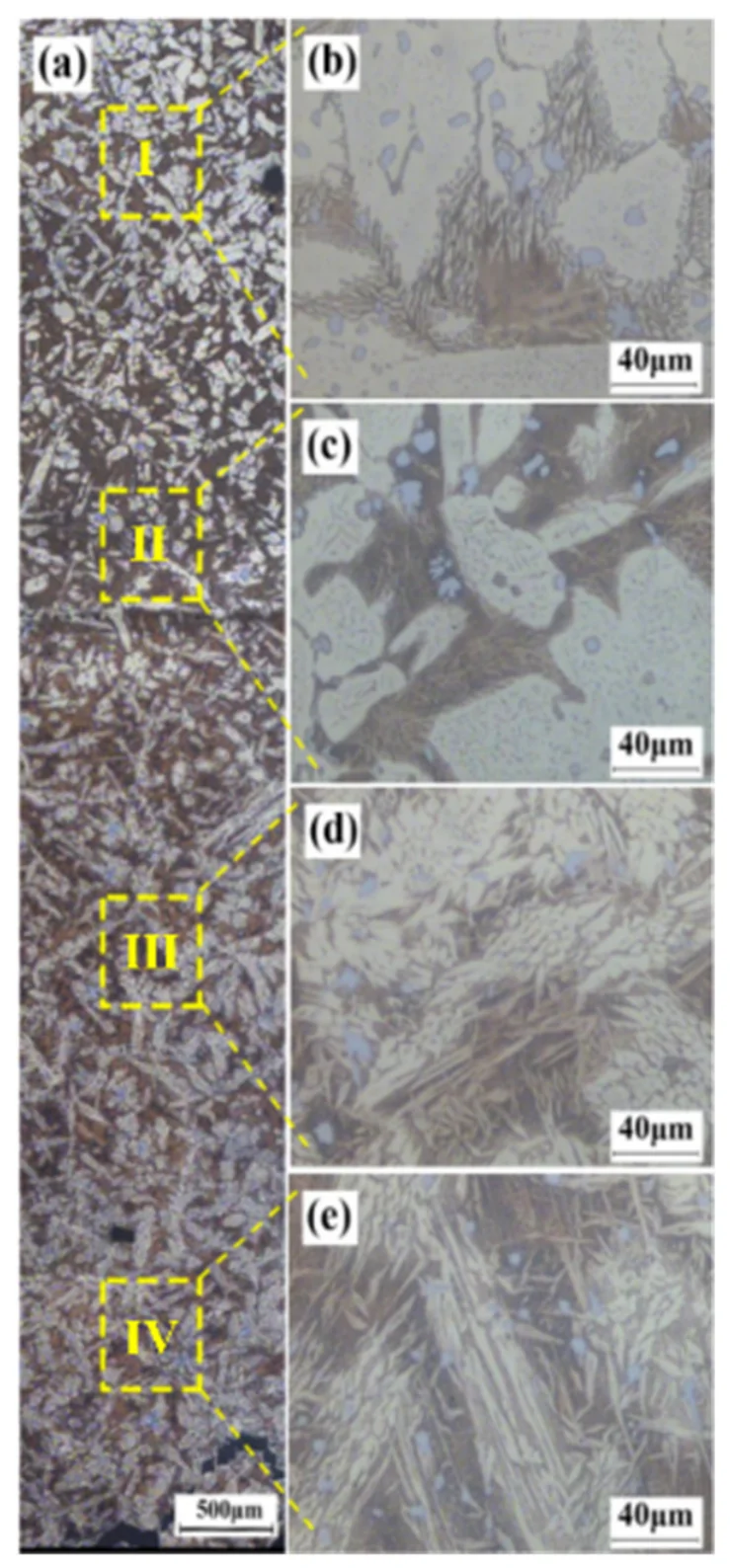
Microstructure of nickel aluminum bronze afterelectropulsing treatment: (**a**) overall morphology; (**b**) enlarged view of area I; (**c**) enlarged view of area II; (**d**) enlarged view of area III; (**e**) enlarged view of area IV [54].

Fang et al. [54] observed the microstructure of nickel aluminum bronze subsequent to electropulsing treatment, as depicted in Figure 3. It can be observed from the figure that under the influence of electropulsing (with a current density of 725 A/mm^2^), the ĸ_III_ phase and ĸ_IV_ phase dissolve successively and that the material gradually exhibits a Weinstein structure. The evolution of the microstructure can be divided into four stages: (I) The layer spacing of the ĸ_III_ phase widens and the β’ phase undergoes a phase transformation. (II) The ĸ_III_ phase disappears and a needle-like β’ phase is formed, which indicates that at this time the Joule heating effect causes the material’s temperature to be higher than the eutectic temperature of the β→α+ĸ_III_ reaction. (III) The ĸ_IV_ phase disappears and the α-phase exhibits needle-like tearing, signifying that the material begins to assume a Weinstein structure. (IV) The material has almost completely transformed into a Weinstein structure.

**Figure 4:**
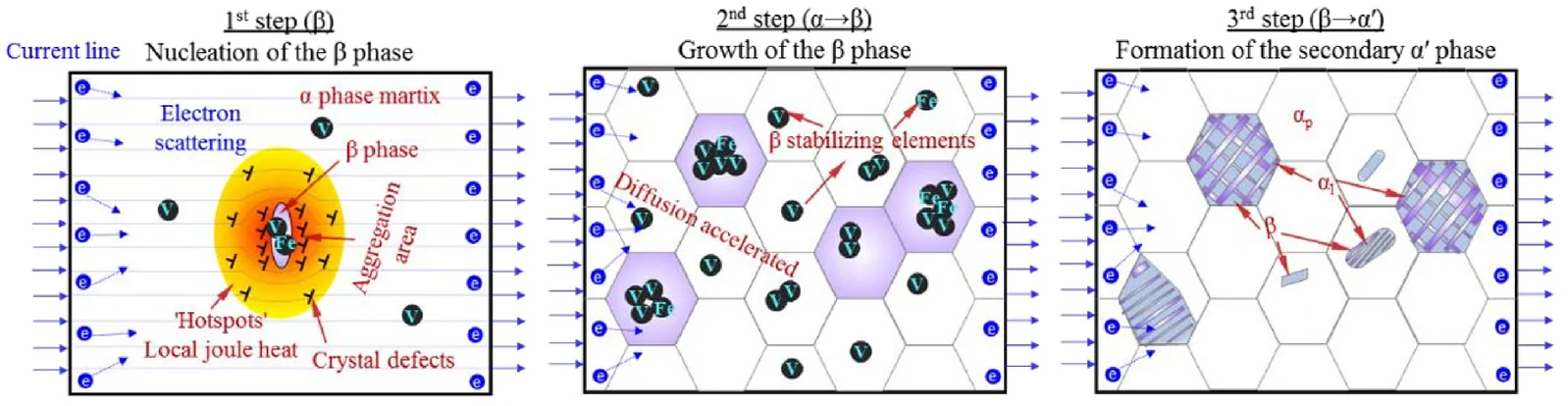
Phase transformation process of the Ti-6Al-4V alloy promoted by electropulsing [55].

Bao et al. [55] investigated the changes in the microstructure of Ti-6Al-4V alloy during a microcompression process assisted by electropulsing (with a current density of 50 A/mm^2^) and put forward a schematic diagram of the phase transformation process of the Ti-6Al-4V alloy promoted by electropulsing, as shown in Figure 4. It can be observed from the figure that the phase transformation of the Ti-6Al-4V alloy is mainly divided into three stages, namely the nucleation stage, the growth stage, and the cooling stage. During the nucleation stage, the temperature of grain boundaries, subgranular boundaries, vacancies, and other defects within the α-phase rises rapidly, which enhances the atomic diffusion rate and triggers the nucleation of the β-phase. During the growth stage, β-phase stabilizing elements such as V and Fe diffuse at a higher rate, leading to the rapid growth of β-phase grains. During the cooling stage, the temperature of defects drops sharply, resulting in the in situ decomposition of the β-phase and the formation of the α’-phase.

#### 2.1.5. Promote Crack Healing

Cracks and micropores will be generated within materials as a result of damage and fatigue during use, which will reduce the service life of materials. Electropulsing can heal the cracks by means of the Joule heating effect [56,57,58,59], promoting atomic diffusion and dislocation movement [58]. Guo et al. [58] observed the microstructure of cracks in TiAl alloys both before and after applying electropulsing in situ. They found that when the pulse current densities were 55 A/mm^2^ and 75 A/mm^2^, the Joule heating effect of electropulsing could effectively promote crack healing. However, when the pulse current density increased to 95 A/mm^2^, the Joule heating effect was so high that it caused thermal damage to the material, which led to the further expansion of cracks.

**Figure 5:**
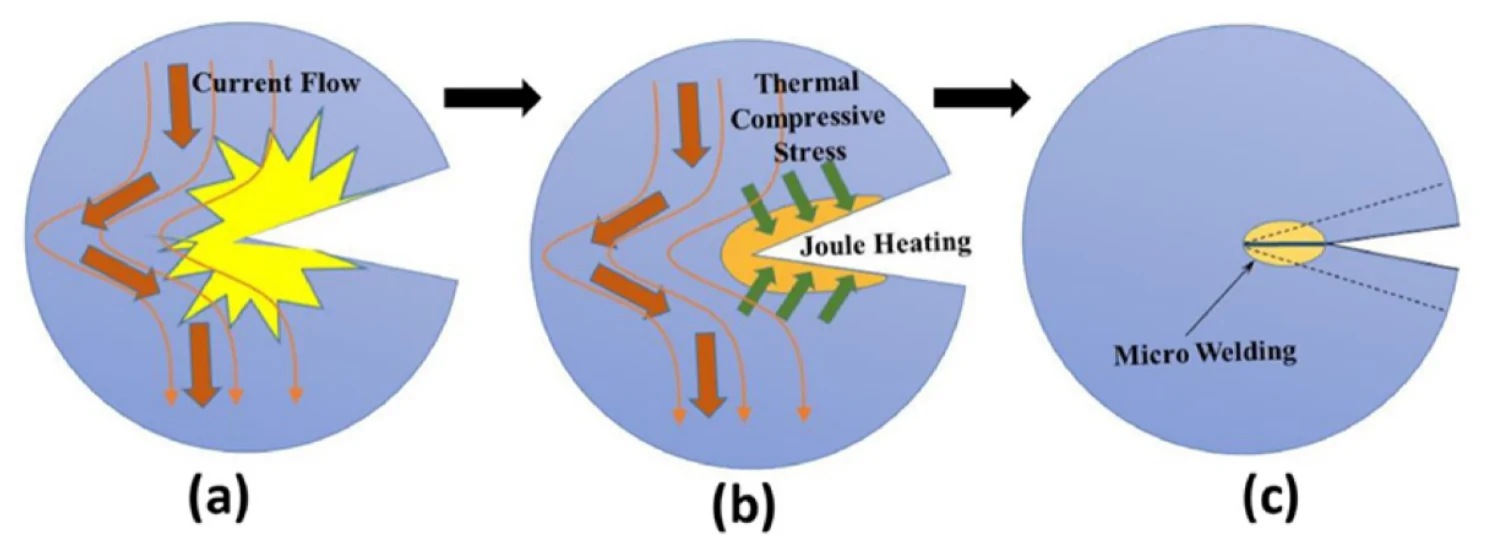
The process of electropulsing promotes crack healing [60]: (**a**) Beginning of electropulsing application; (**b**) during electropulsing application; (**c**) after electropulsing application.

A mechanism and schematic diagram of the way in which electropulsing promotes crack healing is shown in Figure 5. It can be observed from the figure that when electropulsing is applied perpendicular to the crack, the pulse current produces a meandering effect due to the high resistance at the crack tip. This results in a high-intensity electric field being generated near the crack tip, leading to a sharp increase in temperature in that area, while the temperature of other locations rises insignificantly, thus creating a temperature difference with the crack tip. The temperature difference gives rise to an increase in thermo-compressive stress on the crack tip, which prompts the crack tip to start healing. Meanwhile, due to the high dislocation density near the crack, the application of electropulsing accelerates dislocation motion at the tip and on both sides of the crack, facilitating the diffusion of atoms from a high current density region to a low current density region. Subsequently, the atoms on both sides of the crack diffuse into each other under the influence of electropulsing until the crack is completely healed.

#### 2.1.6. Alter Plastic Deformation Mechanism

Electropulsing will enhance the plastic deformation capacity of materials by altering the plastic deformation mechanism, while the plastic deformation mechanism is closely related to material structure. Typically, electropulsing causes significant changes in plastic deformation mechanism within materials that possess an FCC crystal structure. Materials with FCC structure have low stacking failure energy (SFE), which facilitates the formation of stacked laminar dislocations during plastic deformation, resulting in difficult dislocation cross-slip [61]. When materials undergo severe plastic deformation, dislocations are difficult to cross-slip, making them accumulate in plane and form stress concentrations. When the stress reaches critical values, twins are formed in the materials to reduce stress concentration. Twins can induce martensitic nucleation, resulting in a martensitic transformation of material. Therefore, the plastic deformation of the FCC structural materials is predominantly governed by deformation twinning and martensitic phase transformation. However, when appropriate electropulsing is applied during plastic deformation, it can improve the SFE of materials by promoting solute atom precipitation and other mechanisms. This suppresses the formation of stacking faults, facilitates dislocation cross-slip, and consequently shifts the dominant plastic deformation mechanism from twinning and martensitic transformation to dislocation slip.

**Figure 6:**
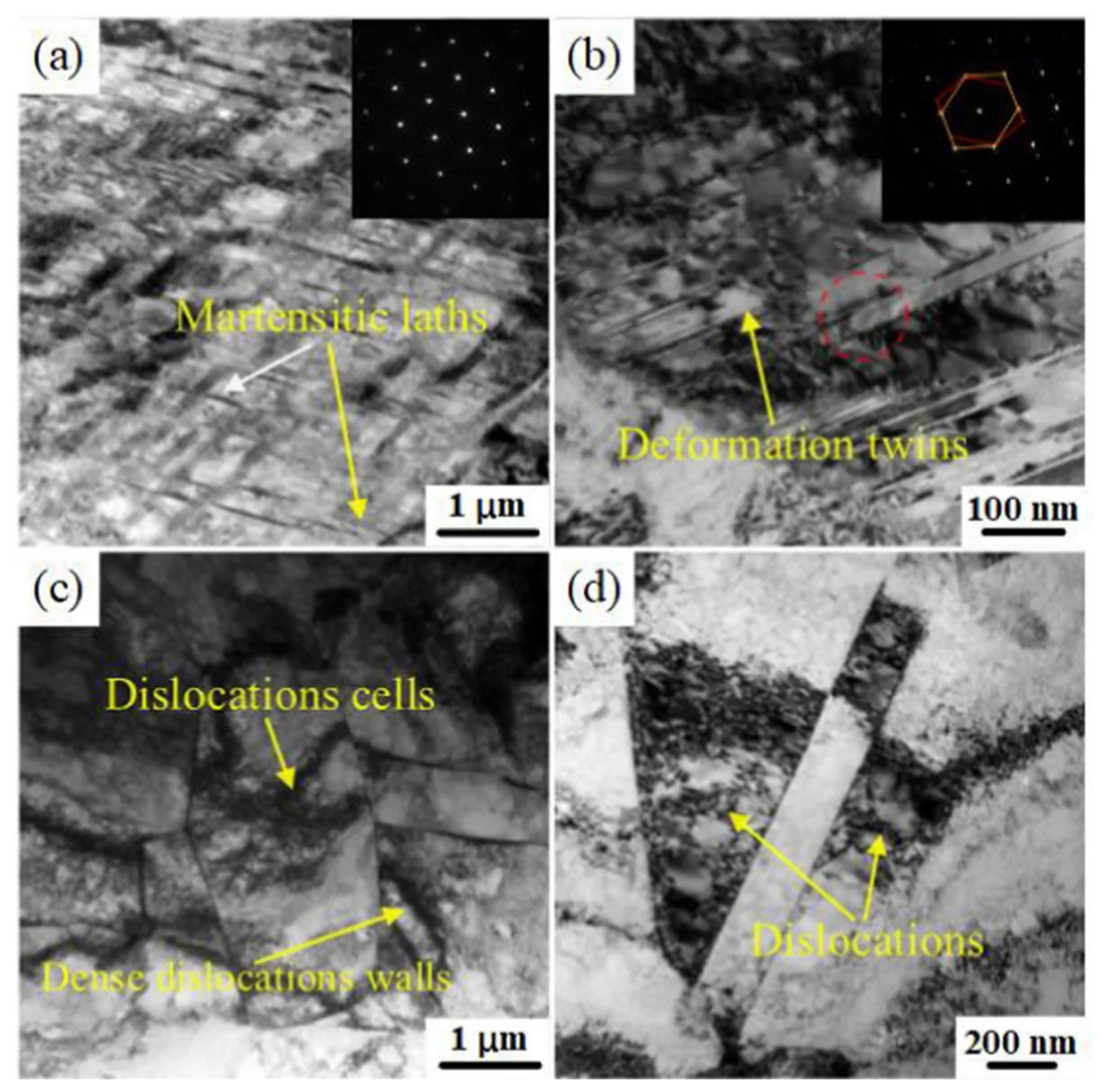
Microstructure of 304 stainless steel after stretching [62]: (**a**,**b**) without applied electropulsing; (**c**,**d**) with applied electropulsing.

Wei [62]. investigated the microstructure of 304 stainless steel following electropulsing-assisted stretching and analyzed the alterations in plastic deformation mechanism, as depicted in Figure 6. It was discovered that the specimens without the application of electropulsing exhibited slaty martensite and twins after stretching, which represents the typical microstructure of 304 stainless steel after plastic deformation at room temperature. However, after the application of electropulsing (with a current density of 133.3 A/mm^2^), slaty martensite and twins were suppressed, and a large number of dislocation cells and dislocation walls were observed instead. This indicated that the plastic deformation mechanism of 304 stainless steel was transformed by electropulsing from one based on deformation twins and martensitic phase transition to one based on dislocation slip.

**Figure 7:**
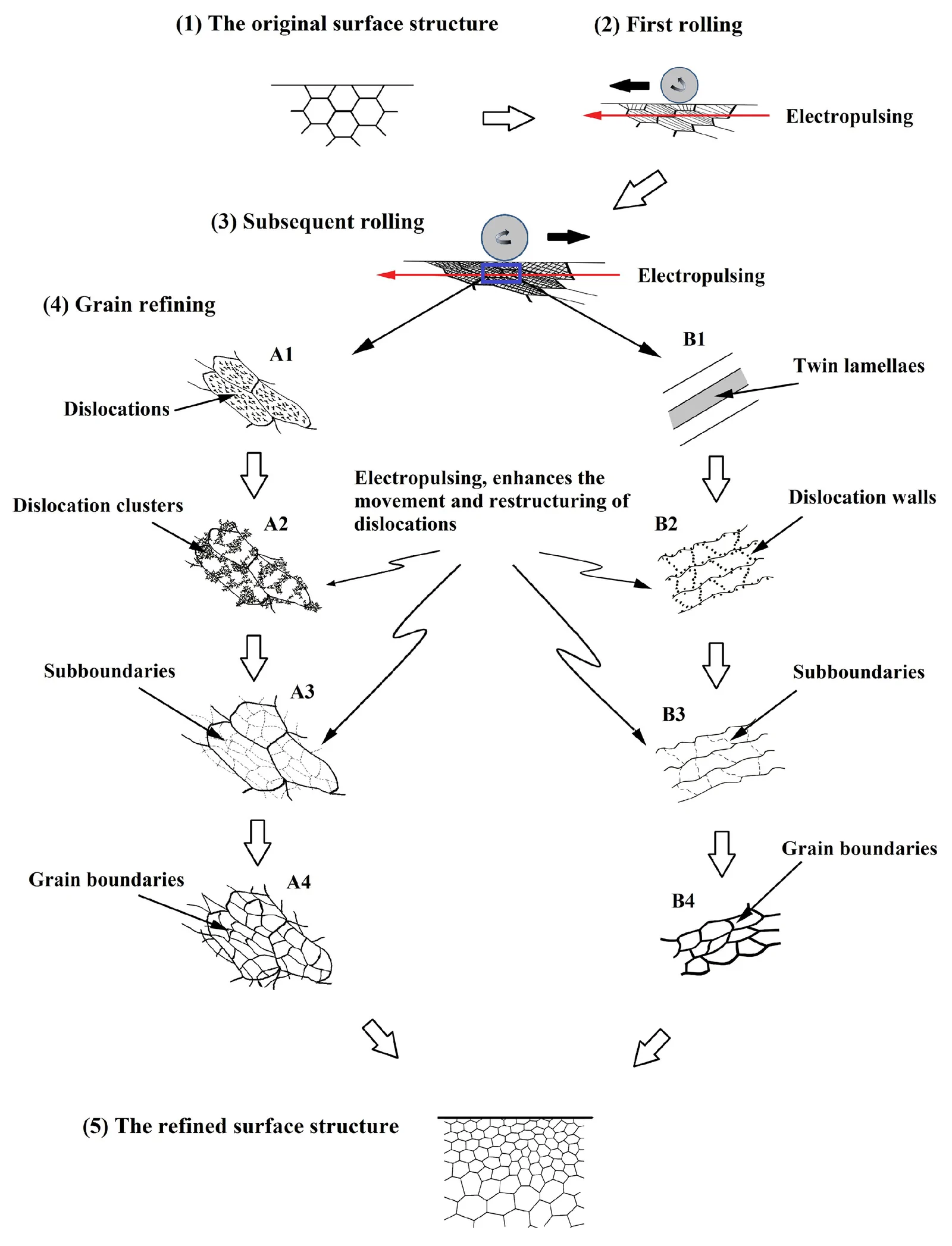
Plastic deformation process of electropulsing-assisted ultrasonic rolling titanium alloy [63].

In addition, Ye et al. [63] analyzed the plastic deformation process of electropulsing-assisted ultrasonic rolling titanium alloy, as shown in Figure 7. It can be seen from the figure that there are two plastic deformation mechanisms in titanium alloy during electropulsing-assisted ultrasonic rolling, which are dislocation slip-based (A1-A2-A3-A4) and deformation twins-based (B1-B2-B3-B4). In the absence of applied electropulsing, the plastic deformation mechanism is dominated by deformation twins but when appropriate electropulsing is applied, the plastic deformation mechanism is dominated by dislocation slip.

In summary, electropulsing can significantly affect the slip system initiation, texture strength, grain size, phase composition, microcrack morphology, and plastic deformation mechanism of the materials. These changes are fundamentally attributed to the thermal and athermal effects of pulsed electric currents, which will be elaborated upon in the following section.

### 2.2. Mechanism of the Effect of Electropulsing on Materials

The mechanism underlying the effect of electropulsing on materials can be principally categorized into the Joule heating effect and the athermal effect. Certain studies contend that Joule heating effect assumes a pivotal role [64,65,66,67,68]. Conversely, other investigations have indicated that the influence of athermal effects are more important [69,70,71]. At present, there remains no definitive conclusion regarding the mechanism of the effect of electropulsing on materials, which is commonly regarded as the combined action of the Joule heating effect and athermal effects [72,73,74]. In this segment, a detailed exposition of the Joule heating effect and athermal effects of electropulsing will be provided.

#### 2.2.1. Joule Heating Effect

Owing to the inherent electrical resistance of metallic materials, when electropulsing is applied to a material, a temperature rise becomes inevitable under the influence of the Joule heat effect [24]. Elevated temperatures result in softening and a reduction in rheological stresses, thereby contributing to the enhanced plastic deformability of the material. The temperature of the material is closely associated with pulse current density, as depicted in Equation (1).

(1) $$\Delta T=\frac{J_{e}^{2}\rho t_{p}}{C_{p}D}$$

where ΔT is the change in material temperature, $J_{e}$ is the effective current density applied on the material or the root mean square current density, ρ is the resistance of material, $t_{p}$ is the width of the pulsed current, $C_{p}$ is the specific heat capacity of the material, and D is the density of the material.

The Joule heating effect is capable of inducing a warming rate of approximately 8 × 10^3^ K/s for metallic materials [75], which plays a significant part in diminishing the rheological stresses [76,77] and augmenting the plastic deformability [65]. Nevertheless, when pulse current is of lower intensity or shorter in duration, the Joule heating effect fails to exert a prominent role, whereas athermal effects wield a greater influence on the plasticity of the material. Cui et al. [78] found that the tensile properties of nickel-based high-temperature alloys assisted by electropulsing could not be accurately predicted by models that took into account only the Joule heating effect or athermal effects. In contrast, the Johnson–Cook (J-C) model, which incorporated both the Joule heating effect and athermal effects, demonstrated the best predictive performance. Consequently, in order to lucidly elucidate the mechanism of the effect of electropulsing on materials, it is imperative to factor in athermal effects in addition to the Joule heating effect.

#### 2.2.2. Athermal Effects—Electron Migration

**Figure 8:**
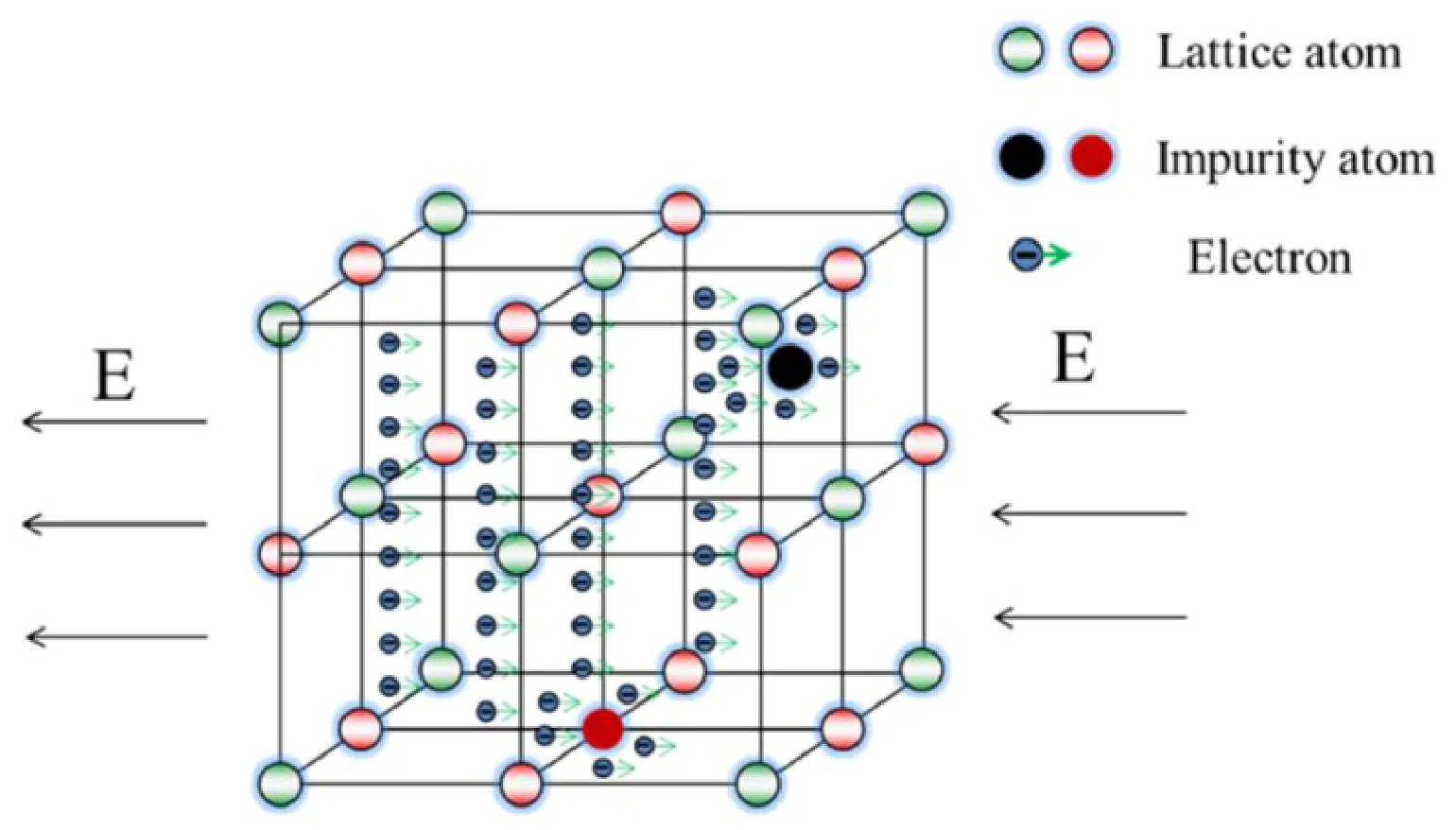
Internal electrons of the material gather at defects under the influence of electropulsing [79].

When electropulsing is applied to metallic materials, the moving electrons excited by pulse current will interact with the internal electrons of the material, causing the internal electrons to accumulate at impurities and defects [79], as depicted in Figure 8. This gives rise to an unbalanced distribution of electrons within the material, facilitating its plastic deformation [63,80]. Similarly, Guo et al. [81] found that the electric field induces the migration of electrons towards grain boundaries and defects, disrupting the charge balance within the material. On the one hand, this diminishes the strength of atomic bonds and enhances the plastic deformation capacity of the material [82]. On the other hand, it magnifies the Joule heating effect and mitigates the impact of athermal effects on the microstructure and properties of the material [83], thereby altering its plastic deformation capacity.

#### 2.2.3. Athermal Effects—Atomic Diffusion

When electropulsing is applied to metal materials, the transient energy generated by electropulsing can be transferred to the interior of the material [84], providing additional free energy for the material system [85,86], enhancing atomic diffusion kinetics [73,87,88,89], and improving atomic diffusion ability within the material. Moreover, electropulsing can effectively reduce vacancy formation energy [90], increase vacancy concentration [91], enhance vacancy activity [92], and promote the diffusion of vacancies [93,94], thereby facilitating atomic diffusion.

In addition, certain research has revealed that phenomena such as electropulsing alter dislocation motion state [95,96], facilitating the precipitation and dissolution of precipitation phases [97,98,99,100], and that induced phase transitions [101,102,103] are all associated with the fact that electropulsing can effectively promote atomic diffusion. Kim et al. [95] explored the electropulsing-assisted tensile process of aluminum alloys and discovered that initial yield stresses of specimens obtained in electropulsing-assisted tensile tests were higher. This is primarily attributable to the fact that a pulsed current enhances the atomic diffusion capacity, which leads to more mobile dislocations being pinned. Gao et al. [104] found that electric pulsing can endow the material with extra Gibbs free energy, giving rise to higher drift fluxes of atoms. Consequently, when compared with conventional heat treatment, the electropulsing treatment can accelerate the dissolution of nanoscale Cu-rich precipitates in aged Fe-Cu alloys. Lu et al. [102] found that at high temperatures, electropulsing promotes atomic diffusion, thereby facilitating the decomposition of the stable δ phase in duplex stainless steels.

In summary, electropulsing is capable of promoting atomic diffusion through augmenting the system free energy, enhancing atomic diffusion kinetics, and reducing vacancy formation energy. Meanwhile, the fact that electropulsing can modify the state of dislocation motion, accelerate the precipitation and dissolution of precipitated phases, as well as facilitate phase transitions, also serves as evidence to prove its can promote atomic diffusion from different perspective.

#### 2.2.4. Athermal Effects—Dislocation Evolution

Regarding the mechanism through which electropulsing promotes dislocation evolution, the electron wind theory put forward by Troitskii et al. [105,106,107,108] is widely acknowledged. This theory asserts that when electropulsing is applied to materials, the moving electrons excited by pulsed current will exert a force on dislocations, propelling them to move. This force is known as the “electron wind force”. The magnitude of the “electron wind force” is closely associated with the pulse current density, as depicted in the Equation (2).

(2) $$F_{ew}=\alpha bp_{f}\left(\frac{j}{e}-n_{e}v_{d}\right)$$

where $F_{ew}$ is the electron wind force, α is a constant with values ranging from 0.5 to 1, b is the Bragg constant, $p_{f}$ is the Fermi momentum, j is the pulsed current density, e is the electron charge, $n_{e}$ is the electron concentration, and $v_{d}$ is the dislocation motion rate. Under the influence of “electron wind force”, the dislocation movement is facilitated, which in turn promotes the dislocation evolution [109]. Additionally, “electron wind force” enhances the lattice vibrational frequency [110] and decreases the activation energy necessary for dislocation slip [111,112], which also contributes to the promotion of dislocation evolution to some extent [113,114].

**Figure 9:**
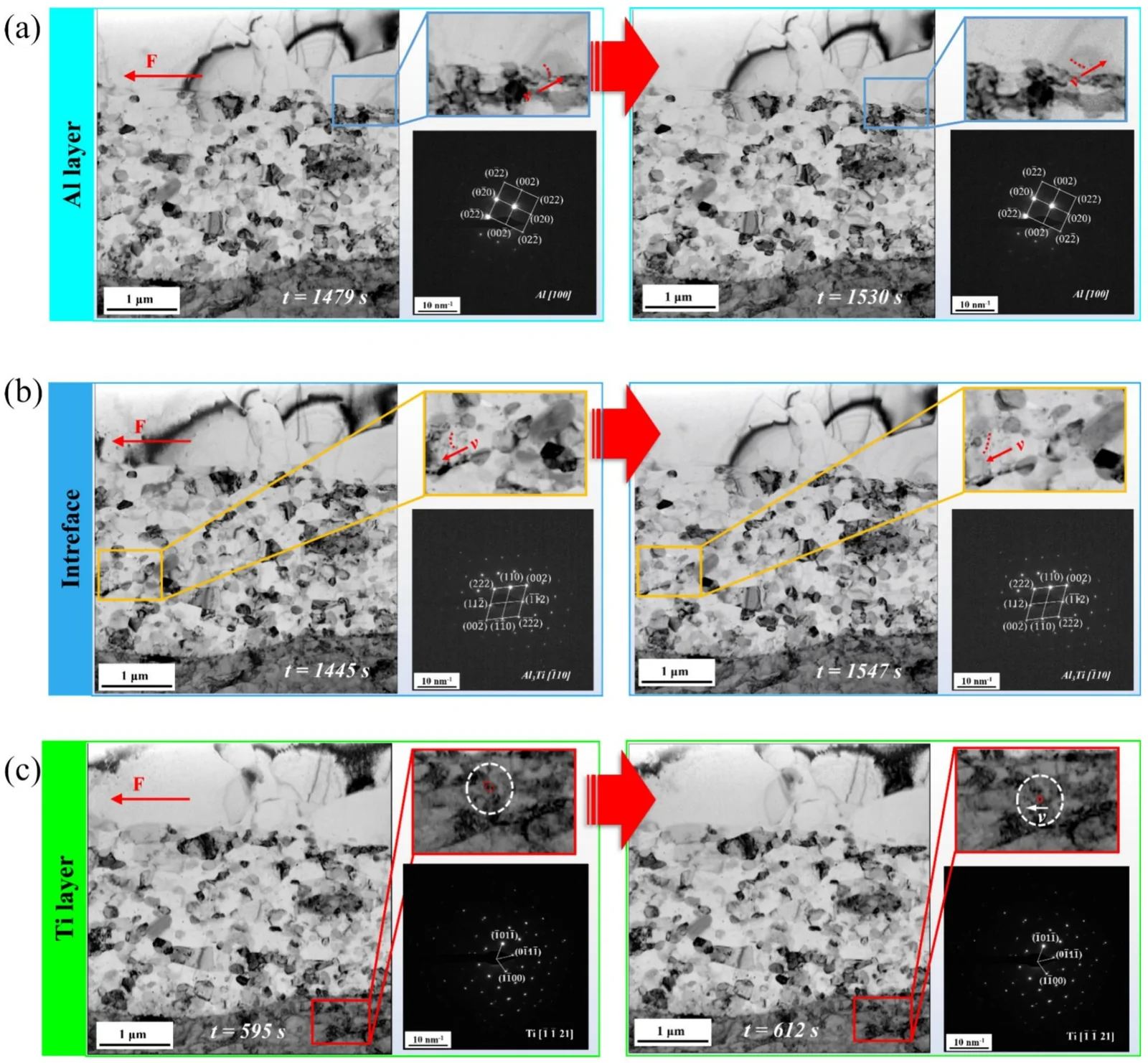
Dislocation evolution driven by “electron wind force” in Ti/Al bimetallic composites: (**a**) Al layer; (**b**) intreface; (**c**) Ti layer [115].

The “electron wind force” is capable of altering the morphology, location, and arrangement of dislocations within the material. Xing [115] observed dislocation evolution driven by the “electron wind force” in Ti/Al bimetallic composites through in-situ transmission electron microscopy, as depicted in Figure 9. From Figure 9, it can be observed that under the influence of “electron wind force”, the positions and morphology of dislocations (marked by red dotted line) are significantly changed. However, in certain cases, the “electron wind force” fails to drive the dislocations, yet it still exerts an influence on the arrangement of dislocations. Xiang et al. [116] discovered through the application of electropulsing to X80 pipeline steel that when the direction of “electron wind force” acting on dislocations is the same as that of drift electrons, the “electron wind force” cannot generate new dislocations from the Frank–Read source, but it will cause the existing dislocations to align parallel to the direction of drift electrons.

When pulse current density exceeds a certain threshold, the “electron wind force” can accelerate dislocation motion, resulting in an increase in dislocation density [117,118,119,120,121]. The mechanical stress exerted on a unit dislocation by drifting electrons in the same direction is shown in Equation (3) [122].

(3) $$F_{e}=\frac{b}{4}\left(\frac{V_{e}}{V_{d}}-1\right)\frac{V_{d}\partial n_{0}}{V\partial \mu }\Delta^{2}$$

where $F_{e}$ is the mechanical stress, b is the Bergner vector, $V_{e}$ is the rate of electron drift, $V_{d}$ is the rate of dislocation motion, $n_{0}$ is the free-carrier density, V is the rate of electron motion in the Fermi surface, μ is the chemical potential, and Δ is the deformation potential constant. When $V_{e}>V_{d}$, dislocations will receive mechanical stress along the same direction of motion, which will cause dislocation motion to be accelerated and dislocation density to be significantly increased.

In addition, the “electron wind force” can also lead to a decrease in dislocation density [37,123,124]. This is primarily attributable to the fact that when an appropriately sized electrical pulse is applied to materials, the dislocation motion will be enhanced by the “electron wind force”. During the process of dislocation movement, second-phase particles, impurities, and other defective particles will impede the dislocation movement, causing dislocations to be pinned and gradually forming dislocation entanglements or dislocation bands as dislocations continue to accumulate. At this time, if the pulse current density is increased, the “electron wind force” will become stronger, resulting in some dislocations breaking away from the pinning [125], reducing the number of dislocation entanglements or dislocation bands [115], and prompting materials to lower the dislocation density through dislocation annihilation or rearrangement (such as the formation of subgrain boundaries) and other means.

**Figure 10:**
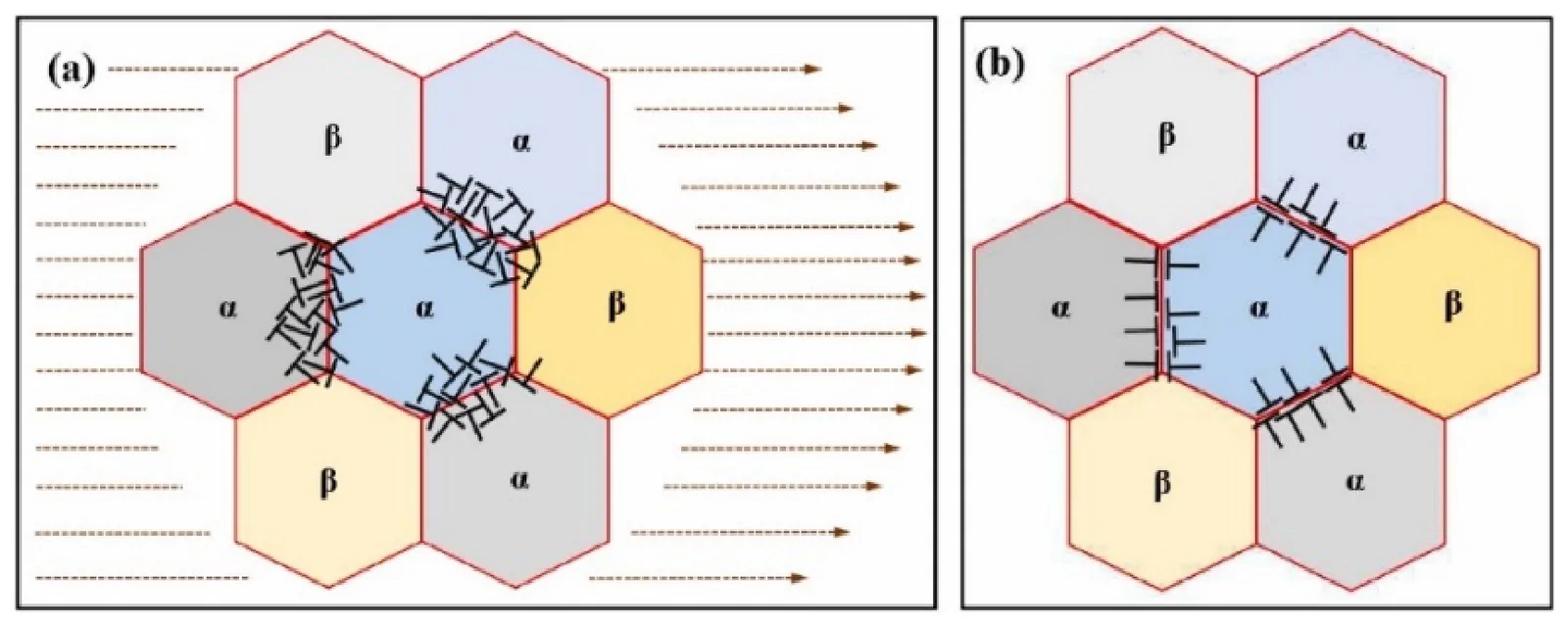
Dislocation evolution near grain boundaries under the effect ofelectropulsings: (**a**) before applying electrical pulse; (**b**) after applying electrical pulse [126].

In order to better illustrate the process by which “electron wind force” reduces the dislocation density, Wang et al. [126] described the dislocation evolution near grain boundaries under the influence of electropulsing, as depicted in Figure 10. It is clearly shown that the distribution of dislocations at the grain boundary is not uniform before the application of electropulsing. A large number of dislocations gather and form dislocation tangles, which is detrimental to dislocation slip. This will lead to stress concentration at grain boundary, ultimately resulting in the formation of microcracks [127] and reducing the plastic deformation capacity of the material. However, when electropulsing is applied, the pulse current enhances atomic motility at the grain boundary, prompting atomic rearrangement at the grain boundary, which in turn leads to the annihilation of some dislocations and the orderly arrangement of the remaining ones.

**Figure 11:**
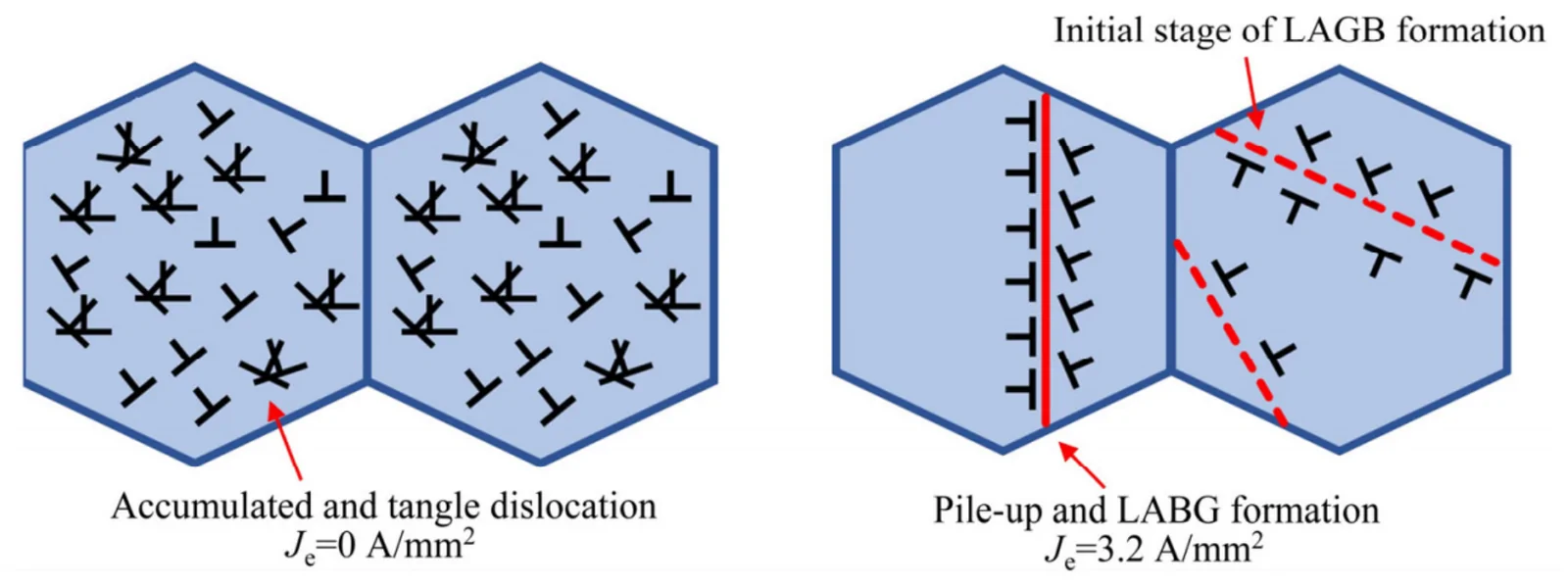
Dislocation evolution within grains under the effect ofelectropulsings [27].

In addition, Meng et al. [27] also observed a similar situation within the grains of Ti-6Al-4V alloy, as shown in Figure 11. Under the influence of electropulsing, the dislocation entanglements were unraveled and some dislocations migrated to the linear regions and gradually accumulated to form subgranular boundaries, thereby reducing the dislocation density of material.

In summary, electropulsing exerts a significant influence on the dislocation evolution process. An appropriate electropulsing is conducive to facilitating dislocation movement, altering the morphology and density of dislocations, and enhancing the plastic deformation capacity of material.

#### 2.2.5. Athermal Effects—Grain Boundary Migration

Through the research presented in the previous two subsections, it has been found that electropulsing can significantly facilitate the motion of atoms and dislocations and it can boost the formation and movement of both small-angle grain boundaries and large-angle grain boundaries [27,128,129], thereby promoting the migration of grain boundaries. Moreover, electropulsing can accelerate the formation of grain boundaries with low interfacial energy [129,130], which renders their migration more straightforward. Zhang et al. [131] discovered that electropulsing can significantly promote the formation of grain boundaries with low interfacial energy in nickel-based high-temperature alloys. This makes it easy to form stacking laminar dislocations within large-angle grain boundaries, thus facilitating the nucleation of annealing twins and leading to an increase in grain boundary mobility.

In addition, recrystallisation serves as the prerequisite for rearrangement of microstructure within materials, and grain boundary migration constitutes one of the key aspects of recrystallisation. Electropulsing can promote recrystallisation by increasing system energy [41], reducing recrystallisation activation energy [37], lowering recrystallisation temperature [132], enhancing nucleation ratio [133], and facilitating the formation of subgranular boundaries [134], thus facilitating grain boundary migration. Yin et al. [128] discovered that electropulsing provided an additional driving force for the recrystallisation of TC11 alloy during plastic deformation, which promoted the recrystallisation of the β-phase at its grain boundaries and dislocation entanglements. Park et al. [135] discovered that electropulsing can reduce the recrystallisation activation energy of AZ31 magnesium alloy by approximately 41%, which effectively promotes the recrystallisation process. Li et al. [42] investigated the electropulsing-assisted recrystallisation process of cold-rolled 2A97 Al-Cu-Li alloy and found that electropulsing could significantly increase the nucleation rate while reducing the recrystallisation temperature. Compared with conventional annealing, electropulsing reduced the recrystallisation temperature from 480 °C to 440 °C and the recrystallisation time from 30 min to 15 s. Gao et al. [136] discovered that electropulsing can promote the movement of dislocations to subgranular boundaries, thereby accelerating the recrystallisation process of hot-rolled TC4 alloys and reducing the recrystallisation time by an order of magnitude.

In summary, electropulsing can remarkably facilitate the migration of grain boundaries via a multiplicity of mechanisms, including facilitating the movement of atoms and dislocations, accelerating the formation of grain boundaries with low interfacial energy, and optimizing the recrystallisation process.

## 3. Electropulsing-Assisted Machining Technology

Compared with traditional processing technologies, electropulsing-assisted machining technology is characterized by an excellent processing effect, high processing efficiency, as well as energy conservation and environmental protection, and has been widely researched in recent years. In the process of electropulsing-assisted machining, the Joule heating effect and the athermal effect of electropulsing work in concert on difficult-to-machine metal materials. This helps to enhance plastic deformation ability, improve microstructure and mechanical properties of materials, and reduce the damage to materials during machining process. Consequently, common electropulsing-assisted machining techniques will be introduced in this section, and their machining effects will be reviewed.

### 3.1. Electropulsing-Assisted Turning

**Figure 12:**
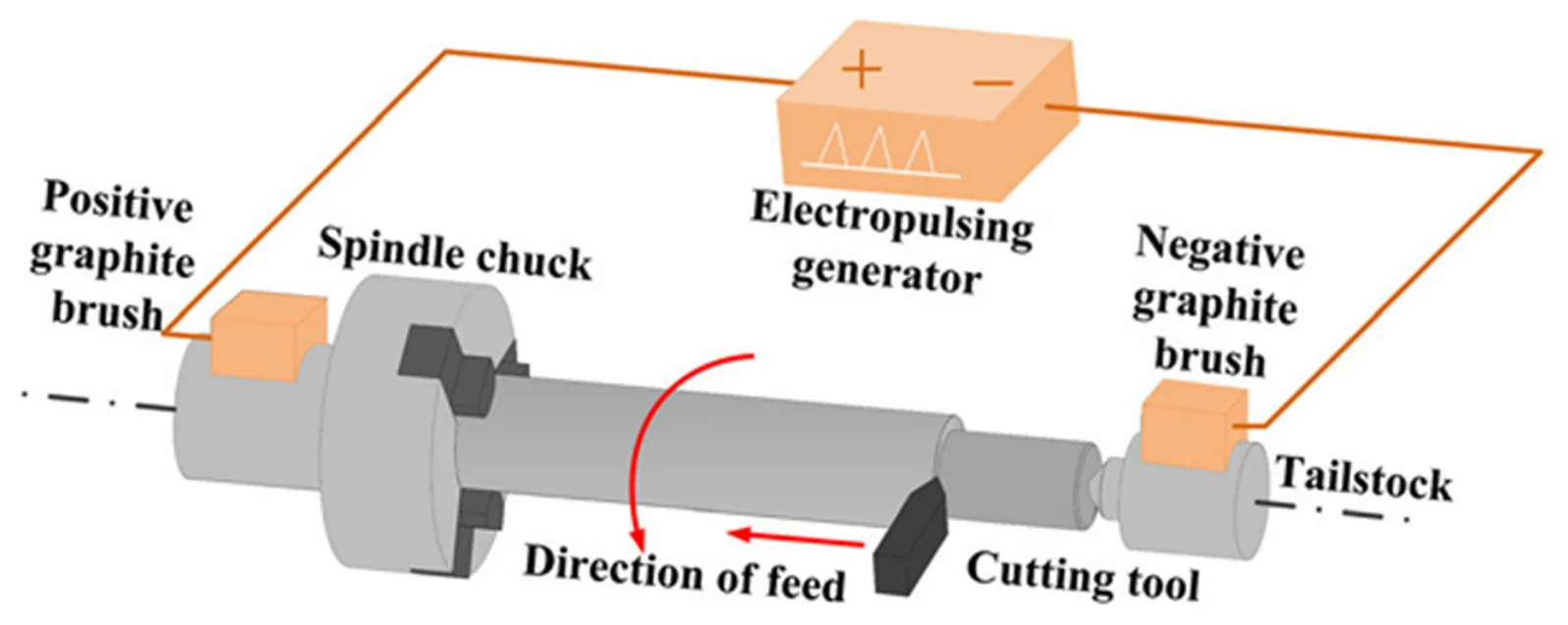
Schematic of electropulsing-assisted turning process [137].

Currently, turning technology has been widely employed in practical production. However, when it comes to the turning of difficult-to-machine metal materials, problems such as severe tool wear and poor machined surface quality always arise. In recent years, researchers have incorporated electropulsing into the turning process, taking advantage of the fact that electropulsing can enhance the plastic deformation capacity of materials to achieve the high-quality turning of difficult-to-machine materials. The schematic diagram of the electropulsing-assisted turning process is illustrated in Figure 12. The electropulsing is generated by an electropulsing generator, the positive graphite brush is connected to the spindle chuck while the negative graphite brush is connected to the tailstock. During machining, the electropulsing is applied to the rotating workpiece, and the tool moves along the direction of workpiece spindle to accomplish turning.

**Figure 13:**
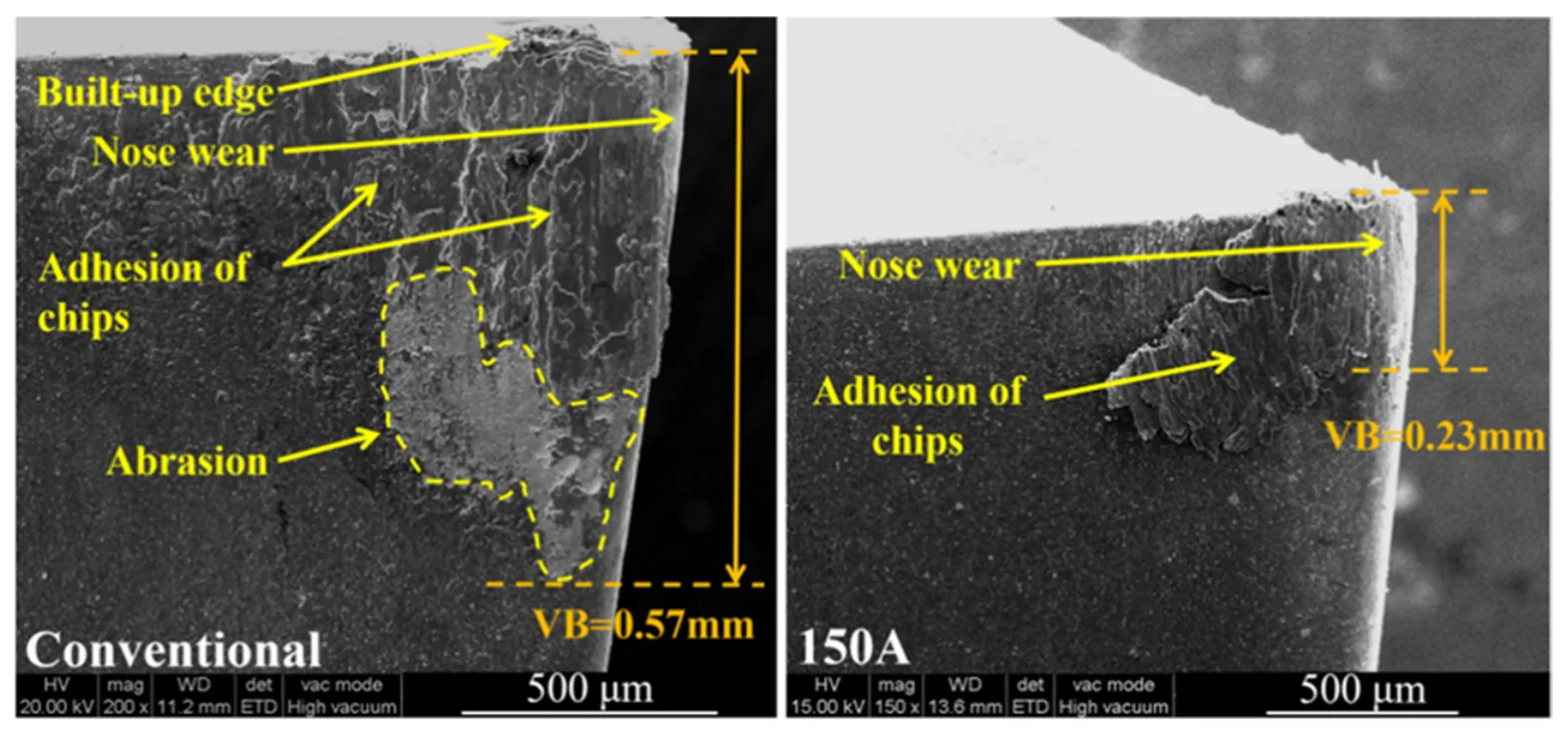
Wear on the flank face of the turning tool [139].

Electropulsing is capable of reducing work-hardening, turning forces, and tool wear during the turning process. Sun et al. [137] carried out electropulsing-assisted turning on a GH4169 high-temperature alloy and found that applying an electropulsing with a frequency of 400 Hz and effective current density of 0.64 A/mm^2^ during the turning process could reduce work-hardening, leading to a reduction of approximately 36.7% in the main turning force. Sánchez-Egea et al. [138] investigated the power consumption during electropulsing-assisted turning of SAE 1045 steel and discovered that, compared with conventional turning, the power consumption of electropulsing-assisted turning was decreased by 104 W and the specific turning energy was reduced by 22%. This was mainly attributable to the significant reduction of turning force in the electropulsing-assisted turning process. Zhai [139] found that electropulsing can promote the plastic flow of surface grains in Ti-6Al-4V, which reduces the turning resistance during machining. As a result, the tangential force, feed force, and radial force were reduced by approximately 20%, 40%, and 40%, respectively. Additionally, applying an electropulsing of 150 A (0.3039 A/mm^2^) at 700 Hz can effectively mitigate the wear on the flank face of the tool, as depicted in Figure 13.

**Figure 14:**
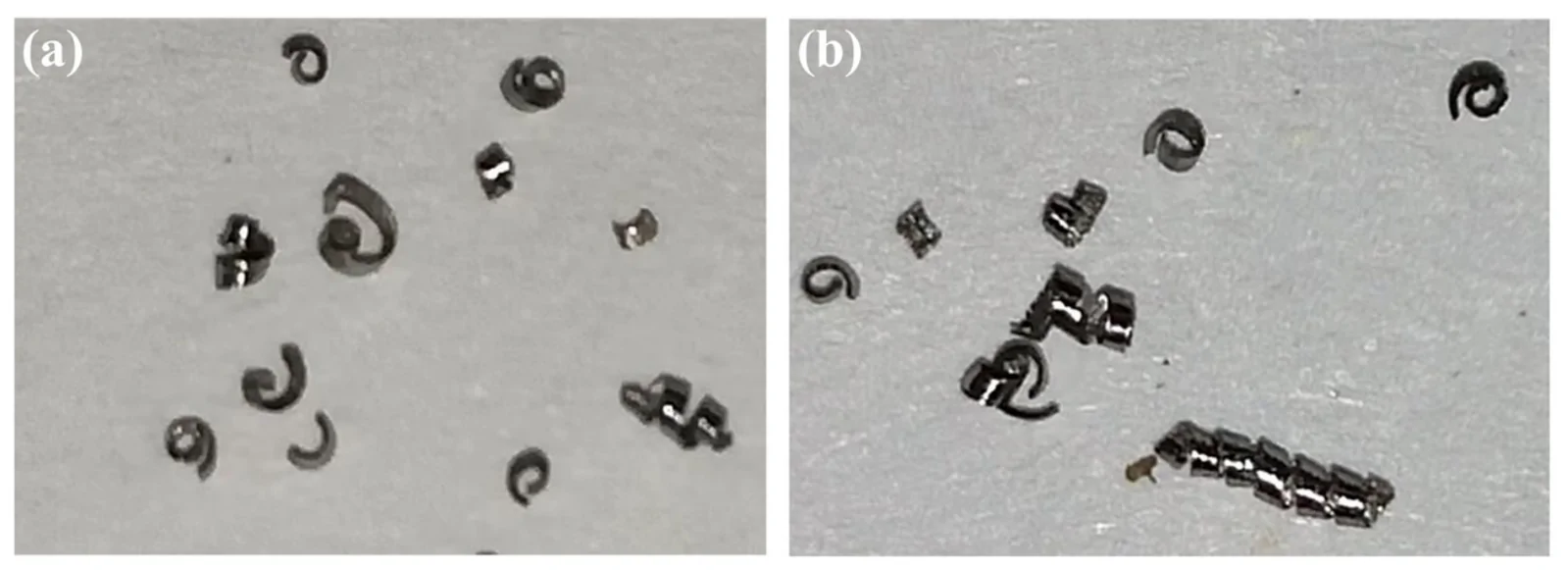
Chip morphology after turning [140]: (**a**) Without electropulsing and (**b**) with 60 V and 110 Hz electropulsing-assisted turning.

Jia [140] observed the morphology of chips from electropulsing-assisted turning, as shown in Figure 14. It can be seen from the figure that the chips from electropulsing-assisted turning are longer and that the pitch and curl radius of the chips are smaller. This indicates that electropulsing makes the turning process easier, also further demonstrating that electropulsing can facilitate the turning process and reduce turning forces and tool wear.

Electropulsing can enhance plastic deformation capability of materials and help to improve the quality of machined surfaces. Wang et al. [18] researched the process of the electropulsing-assisted turning of AISI 304 stainless steel and discovered that electropulsing can make friction between the tool and workpiece into resonance, so that the kinetic energy of friction is converted into heat, causing the turning area to produce melting flow that lubricates the tool and workpiece effectively, resulting in the workpiece being changed from the shear plastic deformation of the traditional turning to plastic stripping and tearing, which effectively improves the surface quality of the machined workpieces. Sun et al. [137] discovered that subgrain boundaries of GH4169 high-temperature alloy increased after electropulsing-assisted turning, resulting in enhanced plastic deformation of the material. Compared with conventional cutting, the surface roughness after electropulsing-assisted cutting was reduced by 74.5%, the surface hardness was reduced by 17.3%, and the depth of the surface deformation layer was increased by two to three times. Zhao [140] et al. researched the effect of electropulsing-assisted turning on the surface roughness of W93NiFe alloy and discovered that when the pulse voltage is lower than 70 V, there is no significant change in the surface roughness of the material after electropulsing-assisted turning; when the pulse voltage is 80 V, the surface roughness of the material after electropulsing-assisted turning decreases significantly, with a maximum reduction of 38.94% compared with traditional turning; however, when pulse voltage increased to 90 V, the high pulse voltage will lead to an increase of 29.63% in the surface roughness value of the material. Xu et al. [141] observed the surface morphology of AISI 5120 steel after electropulsing-assisted turning. The protrusion between cutting grooves of the sample without applied electropulsing is clear, and is mainly due to the poor plastic deformation ability of the sample, resulting in discontinuous chips and leading to poor machining surface quality. With the increase of pulse current density, the protrusion between cutting grooves gradually reduces, and the machined surface quality of samples gradually increases. However, when the current density is 1.1 A/mm^2^, the pulse current leads to a sharp increase in the temperature of the samples, which makes the tool wear serious, and ultimately leads to a decline in the machined surface quality of samples.

**Figure 15:**
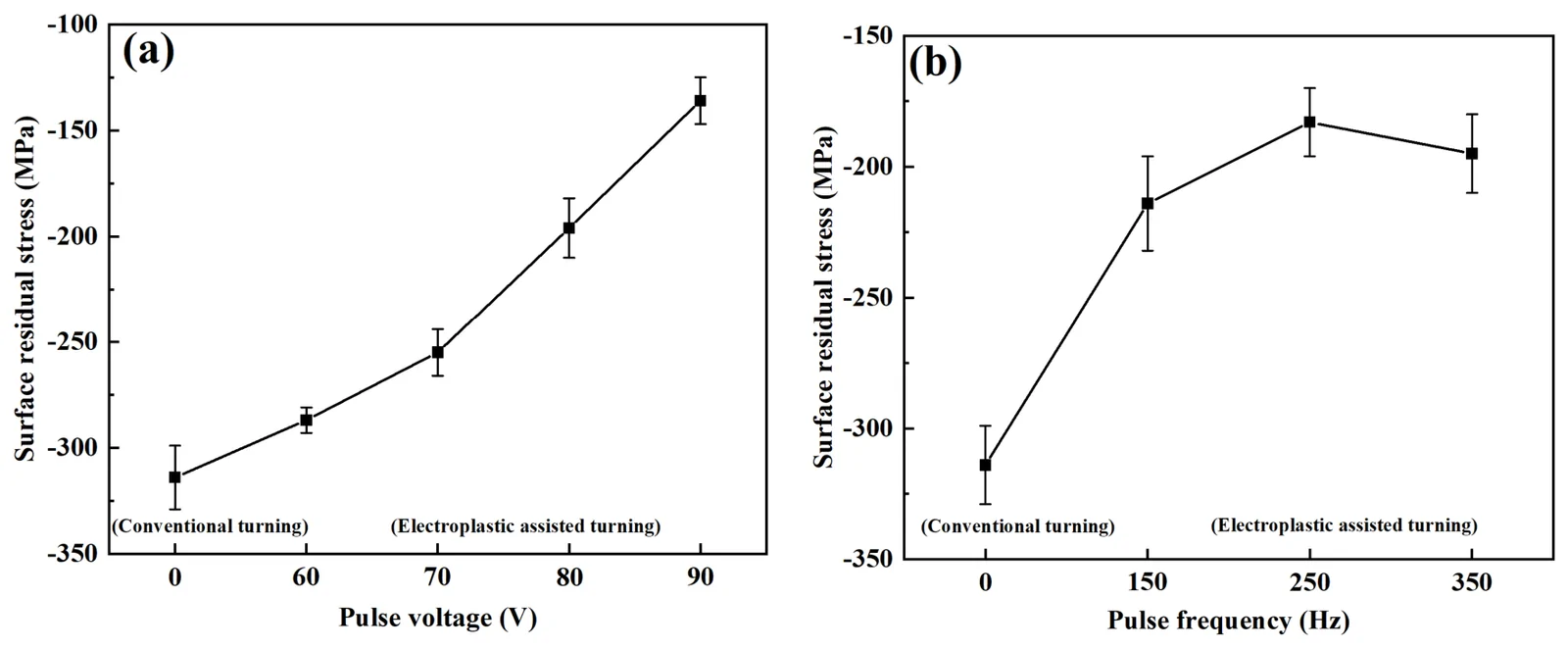
Effect of different parameters of electropulsing-assisted turning on internal residual stresses in W93NiFe alloy [142]: (**a**) pulse voltage and (**b**) pulse frequency.

Electropulsing-assisted turning can reduce the residual stresses inside materials after turning. Liu et al. [142] investigated the residual stresses in W93NiFe alloy after electropulsing-assisted turning, as shown in Figure 15. It was found that the residual stresses inside materials were reduced after electropulsing-assisted turning when compared with conventional turning. This is mainly due to the fact that electropulsing promotes the dislocation movement and reduces the internal dislocation density of the material, which reduces the lattice distortion of materials during plastic deformation, and thus reduces the internal residual stresses in materials.

In summary, electropulsing helps to improve the plastic deformation capacity of materials, reduce the cutting force in turning process and the residual stress inside materials after turning, reduce tool wear, improve the quality of turning surface, and achieve the high-quality turning of difficult-to-machine metal materials.

### 3.2. Electropulsing-Assisted Drawing

**Figure 16:**
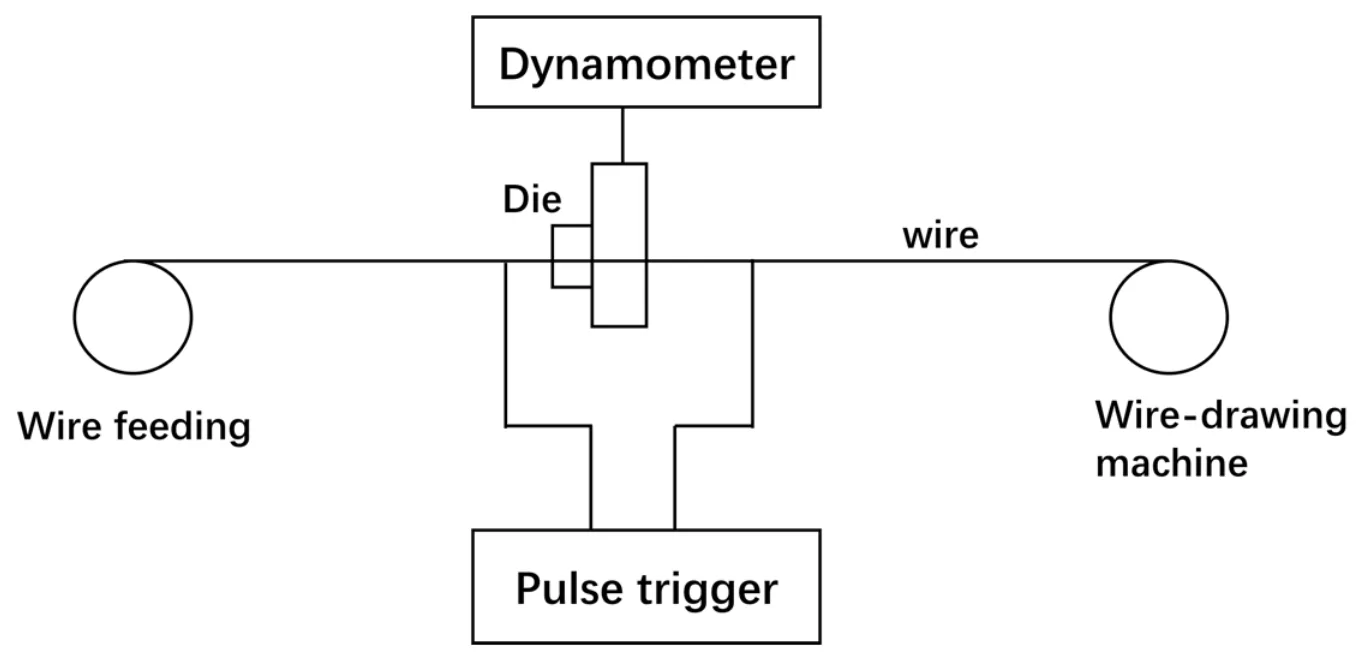
Schematic of electropulsing-assisted drawing process [122].

Conventional drawing technology has been widely employed in the production of tubes, bars, and wires to bring about simultaneous changes in the diameter and thickness of materials. This process boasts high processing efficiency and is highly suitable for the production of materials with greater lengths and smaller diameters. In terms of difficult-to-machine metal materials, annealing and pickling are necessary during the drawing process, which complicates the process. Moreover, due to the poor deformation ability of difficult-to-machine materials, they usually need to be drawn repeatedly, thereby reducing the processing efficiency. Additionally, the drawing force of the drawing process is considerable, which tends to cause damage to the material’s surface, thus affecting the processing quality. However, if electropulsing is incorporated into traditional drawing process, it can enhance the plastic deformation ability of materials and contribute to simplifying the processing process, reducing the drawing force, and improving both the processing efficiency and quality. The schematic diagram of the electropulsing-assisted drawing process is shown in Figure 16. In this process, electropulsing is applied before and after the drawing die, the drawing force tester is connected to the drawing die, and the material is drawn by a specialized drawing machine.

Electropulsing can reduce the degree of work-hardening and significantly reduce the drawing force during drawing process. Tang et al. [143] discovered through the process of electropulsing-assisted drawing of 304 stainless steel that the drawing force during the electropulsing-assisted drawing process was reduced by about 20%~50% when compared with the conventional drawing process. This is mainly because the electropulsing provides additional energy to the material, enabling dislocations to effectively overcome obstacles in slip surface, in turn reducing the degree of work-hardening and the deformation resistance. Furthermore, electropulsing can affect friction factor by influencing the electrons on the surface of the material, so that the internal and external friction of the material can be reduced, which leads to the reduction of drawing force. This reduction in drawing force eliminates the need for annealing during the drawing process and greatly improves the processing efficiency.

**Figure 17:**
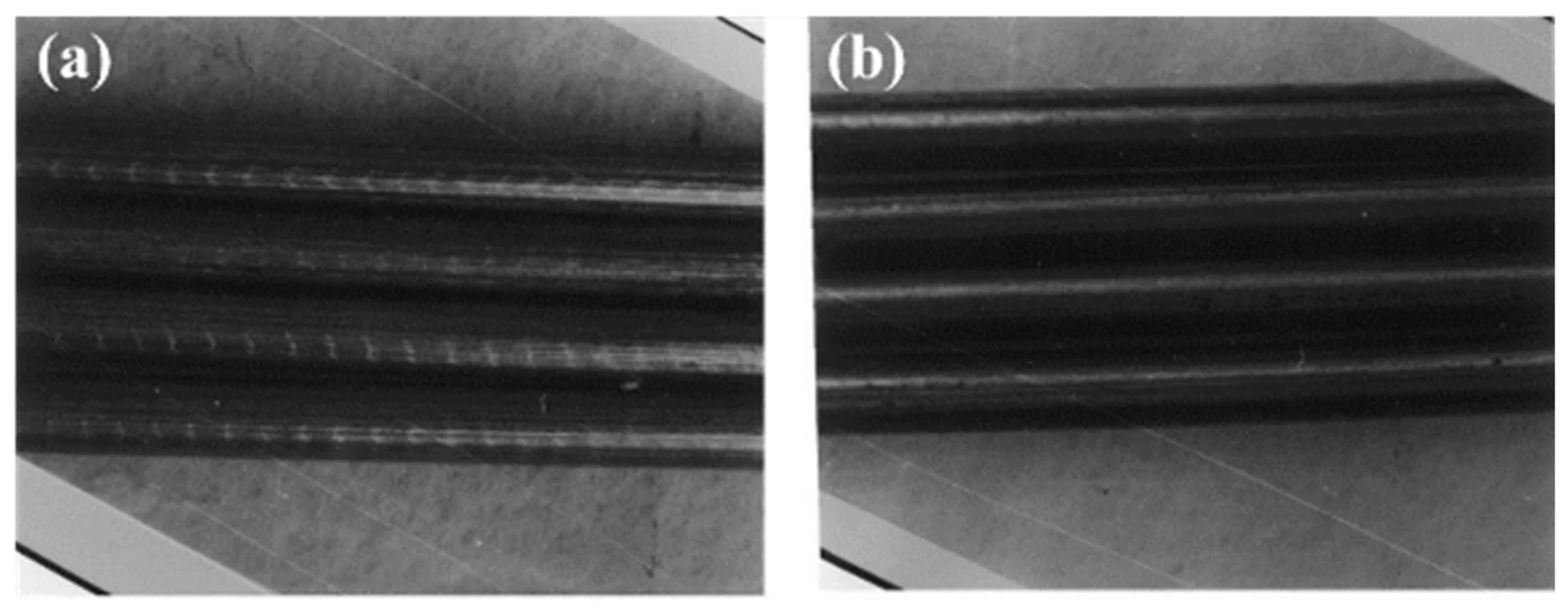
Surface morphology of 304 austenitic stainless steel after drawing [137]: (**a**) Conventional drawing and (**b**) electropulsing-assisted drawing.

Electropulsing can enhance plastic deformation capacity of materials, thus improving the surface quality of materials after drawing. Tang et al. [144] observed the surface morphology of 304 austenitic stainless steel after electropulsing-assisted drawing, as shown in Figure 17. It can be seen from the figure that the surface quality of materials after traditional drawing is poor, there are many ‘knots’ on the surface, but the surface quality of materials after electropulsing-assisted drawing is significantly improved. This is mainly due to the poor plastic deformation of materials during the conventional drawing process, where a large number of dislocations are pinned by grain boundaries or second-phase particles, leading materials to undergo martensitic phase transformation and resulting in the hardening of materials and processing difficulties, ultimately forming ‘knots’ on the surface of materials. However, electropulsing makes the plastic deformation mechanism of materials change from a deformation-induced martensitic phase transformation to dislocation slip, effectively improving the plastic deformation capacity of materials, reducing the degree of work-hardening, and improving the quality of machined surfaces.

Electropulsing-assisted drawing can also change the mechanical properties of materials. Tang et al. [144] summarized the changes in the mechanical properties of 304 austenitic stainless steel after electropulsing-assisted drawing, as shown in Table 1. It can be seen from the table that the tensile strength of materials decreases and that elongation increases after high-energy electropulsing-assisted drawing when compared with conventional drawing techniques. This is mainly because the content of martensite inside materials after traditional drawing is higher, which makes the strength of the materials higher and their elongation lower. However, electropulsing makes the content of martensite inside materials lower, while, at the same time, electropulsing can promote dislocation movement, giving materials lower strength and higher elongation after electropulsing-assisted drawing. In addition, Tang et al. [122] found this phenomenon in the electropulsing-assisted drawing 304L austenitic stainless steel, the ultimate tensile strength of 304L austenitic stainless steel decreased about 13%~35% after electropulsing-assisted drawing.

In summary, electropulsing-assisted drawing can reduce work-hardening, improve plastic deformation capacity, reduce drawing force, save annealing procedure in traditional drawing, enhance machining efficiency, improve the quality of machined surfaces, and increase the elongation of materials, giving it a broad prospect for application in the engineering field.

### 3.3. Electropulsing-Assisted Rolling

**Figure 18:**
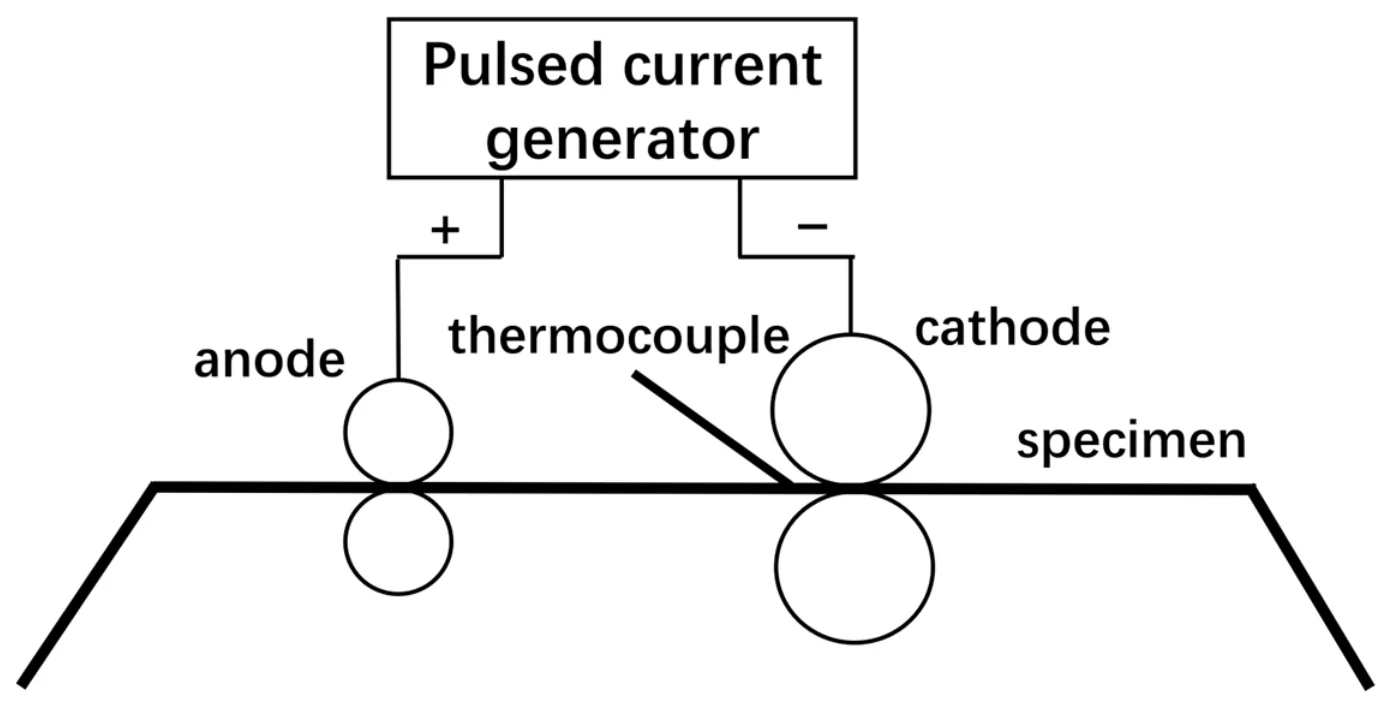
Schematic of electropulsing-assisted rolling process [30].

Rolling as a mature metal material processing technology has been extensively utilized in industrial production. This technology can directly reduce the thickness of materials without additional processing and endows materials with superior overall properties. However, when dealing with difficult-to-machine metal materials, the material typically experiences severe work-hardening. This phenomenon increases the deformation resistance, making rolling arduous. Consequently, heat treatments such as annealing or recrystallization must be implemented prior to further rolling, resulting in a complex process [145]. When electropulsing is introduced into the conventional rolling process, electropulsing can enhance the plastic deformation capacity of difficult-to-machine metal materials and decrease the deformation resistance, thereby improving the rolling quality [146]. The schematic diagram of electropulsing-assisted rolling process is presented in Figure 18. Electropulsing is generated by a pulse current generator, the large roller serves as the negative pole, and the small roller serves as the positive pole, thermocouples are employed in the vicinity of a large roller to monitor the temperature change of the material surface. As can be observed from the figure, the thickness of material is significantly reduced after passing through large roller, indicating that the plastic deformation of material mainly takes place at large roller.

Electropulsing can reduce the deformation resistance of materials, which leads to a significant reduction in rolling force during rolling process. Wang et al. [147] investigated the variation of rolling force during electropulsing-assisted rolling and the conventional hot rolling of AZ31 magnesium alloy, as shown in Table 2. It can be seen from the table that the two rightmost columns indicates the rate of rolling force reduction, meaning that the larger the value is, the clearer the reduction of the rolling force is. When the rolling temperature is the same, the rolling force reduction of electropulsing-assisted rolling is significantly higher than that of conventional hot rolling. Lu et al. [148] compared the changes in rolling force during electropulsing-assisted rolling and conventional rolling of Bi-2333/Ag and discovered that the average reduction in rolling force during electropulsing-assisted rolling was about 14.3%, and the maximum reduction was about 21%.

**Figure 19:**
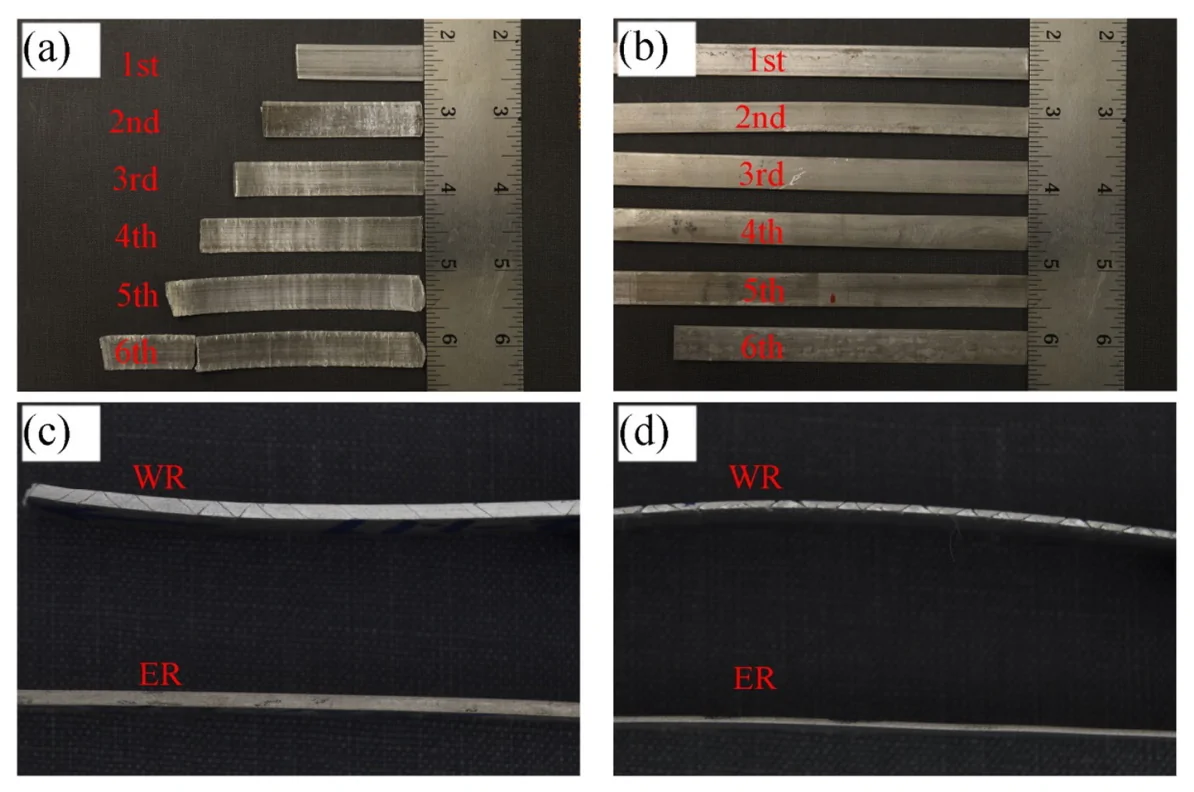
Surface morphology of AZ31 magnesium alloy after rolling [30]: (**a**) Conventional warm rolling; (**b**) electropulsing-assisted rolling; (**c**) 2nd rolling; and (**d**) 6th rolling.

Electropulsing can improve the plastic deformation capacity of materials and reduce the deformation resistance of materials, resulting in a significant increase in machining quality. Kuang et al. [30] compared the surface morphology of AZ31 magnesium alloy plate with the same thickness after conventional warm rolling (WR) and electropulsing-assisted rolling (ER), as shown in Figure 19. It can be seen from the figure that the conventional warm rolled samples began to crack at the edge after the 2nd rolling, and that the samples were completely fractured after the 6th rolling; while the electropulsing-assisted rolling samples did not have cracks and fractures after the 6th rolling and the rolling quality was significantly improved.

**Figure 20:**
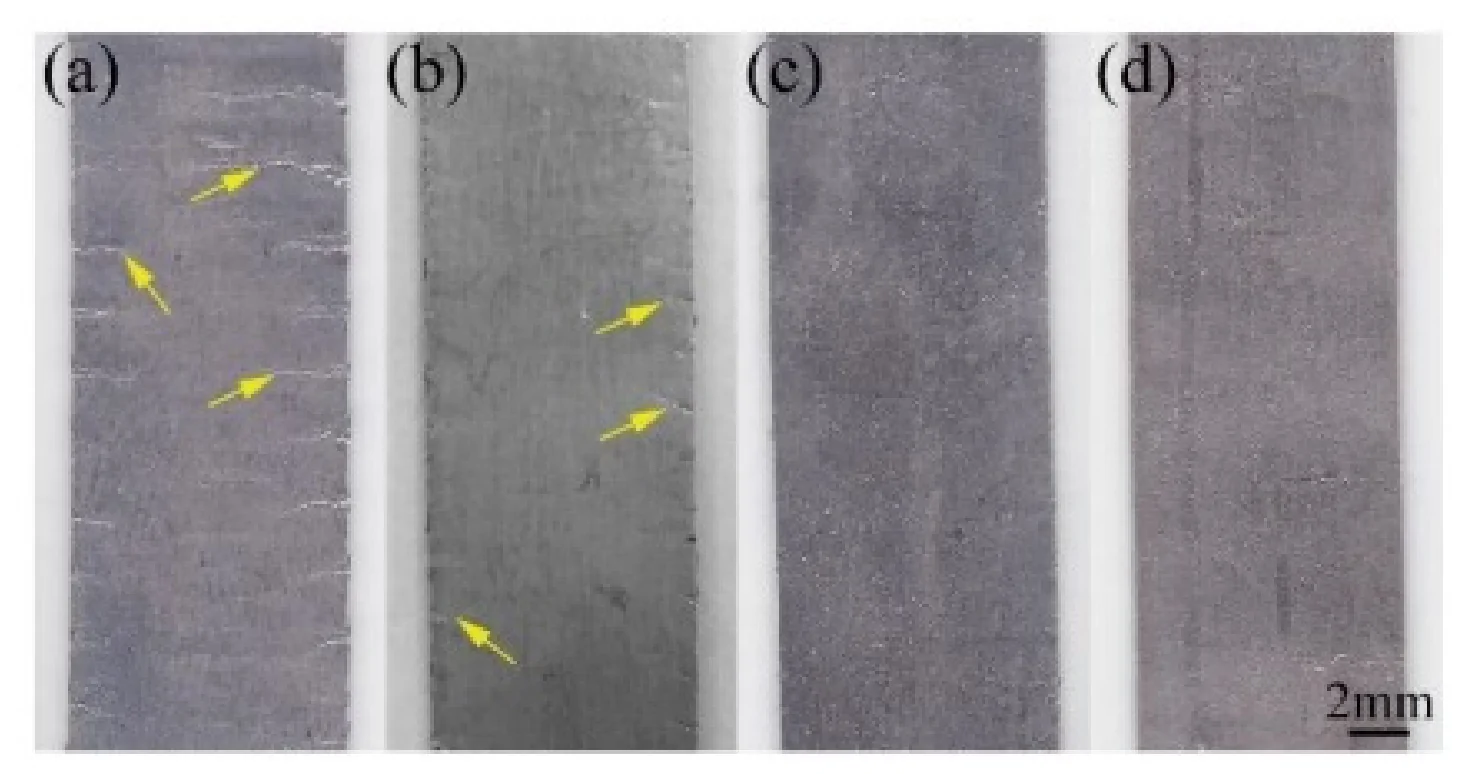
Surface morphology of AZ31B magnesium alloy after rolling by different processes [149]: (**a**) Cold rolling; (**b**) rolling at 60 °C; (**c**) rolling at180 °C; and (**d**) 6300 A, 500 Hz, 70 μs electropulsing-assisted rolling (56.3 °C).

Sun et al. [149] investigated the effect of electropulsing on surface edge cracking behavior of AZ31B magnesium alloy during rolling process, and it was found that electropulsing can effectively inhibit the surface edge cracking (pointed by the yellow arrow) of AZ31B magnesium alloy strips during the rolling process, as shown in Figure 20. Electropulsing-assisted rolling reduces the grain size and increases the number of shear bands, which reduces the strain on single shear bands and has positive effects on inhibiting crack formation. Additionally, electropulsing promotes dynamic recrystallisation and improves the plastic deformation capacity of materials, which also effectively inhibits crack generation.

**Figure 21:**
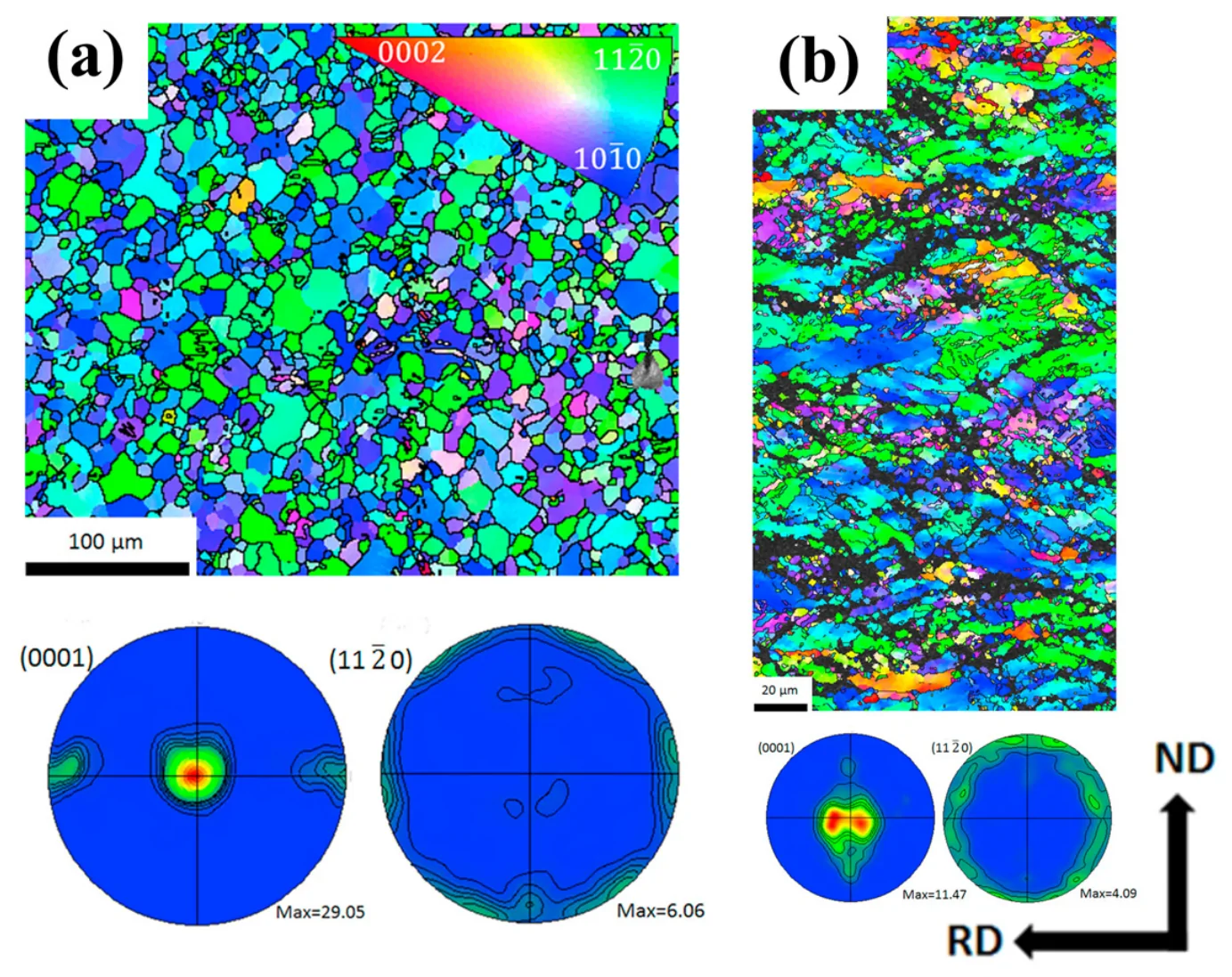
Microstructure of AZ31 magnesium alloy before and after electropulsing-assisted rolling [150]: (**a**) Before rolling and (**b**) after rolling.

Electropulsing-assisted rolling can cause significant changes in the microstructure of materials. Li et al. [150] investigated the microstructural changes of AZ31 magnesium alloy before and after electropulsing-assisted rolling, as shown in Figure 21. The material before rolling consists of fully recrystallised grains with an average grain size of about 10 μm and the texture strength in the material is large. However, the grain is elongated after rolling and many large deformed grains with irregular grain boundaries are formed, there are also a large number of shear bands inside grains, while the texture strength of the material is reduced.

**Figure 22:**
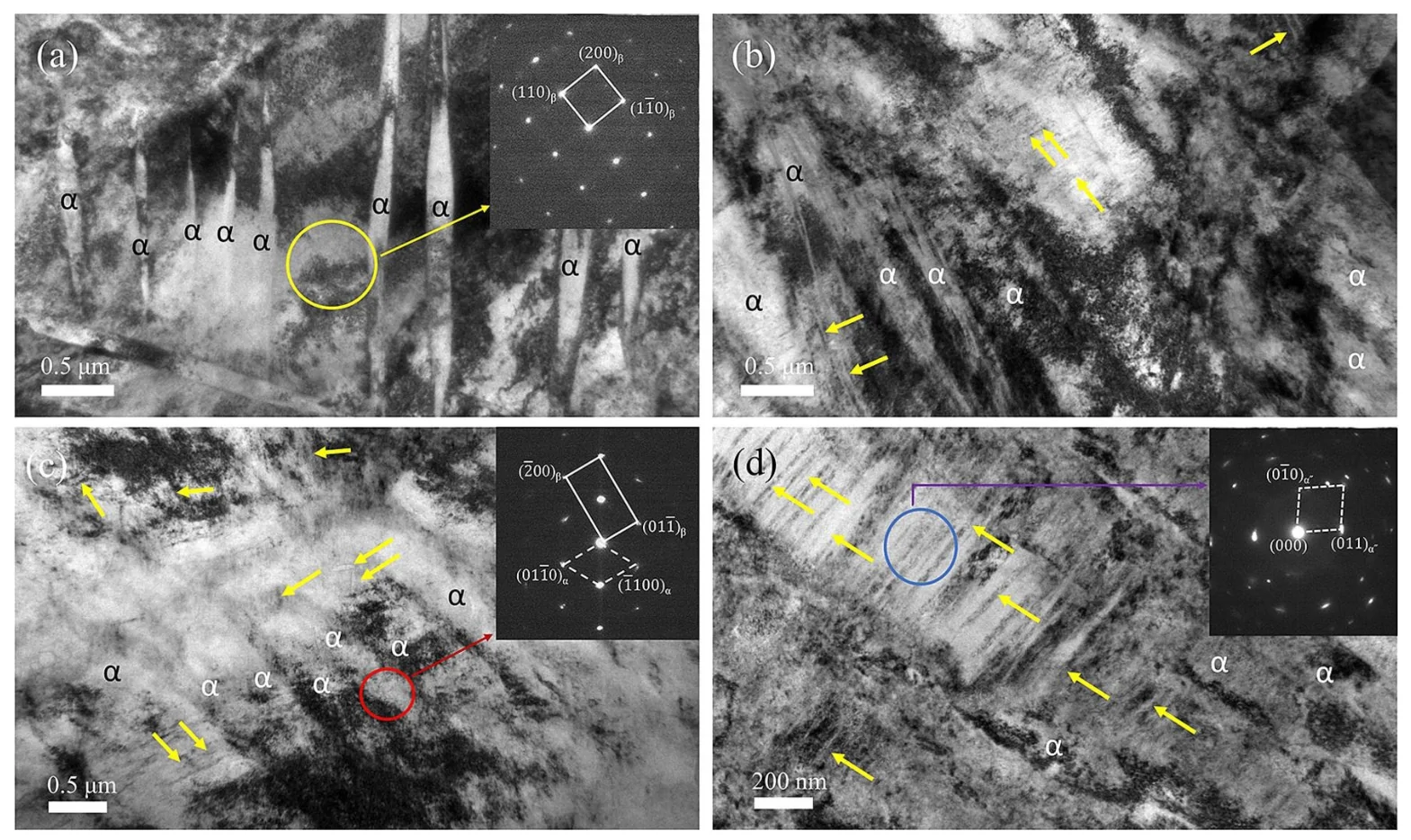
Microstructure of TiZr alloy after electropulsing-assisted rolling [151]: (**a**) Strain 1.31; (**b**) strain 1.74; (**c**) strain 2.28; and (**d**) strain 2.70.

Deng [151] investigated the microstructural changes of TiZr alloy after electropulsing-assisted rolling, as shown in Figure 22. When the strain was 1.31, slaty α-phase started to appear in the β-phase and the sample started to undergo martensitic transformation. When the strain increased from 1.74 to 2.28, the β-phase gradually decreased and the α-phase gradually increased in the sample, in addition there were many α”-phase sheets that appeared in the α-phase (yellow arrows in Figure 22). Meanwhile, when the strain increased to 2.70, the martensitic transformation was completed in the sample, and the β-phase was completely transformed into α-phase and α”-phase.

In addition, electropulsing-assisted rolling can affect the mechanical properties of materials. Qian et al. [152] discovered that the strength and hardness of AISI 1010 low-carbon martensitic steel after electropulsing-assisted rolling was slightly lower than that of cold-rolled specimens and that the elongation was higher than that of cold-rolled specimens at the same amount of deformation (30%). Table 3 summarizes the mechanical properties of several difficult-to-machine metal materials after electropulsing-assisted rolling, from which it can be seen that electropulsing-assisted rolling has little effect on the tensile strength of materials, but has a significant effect on the elongation of materials.

In summary, the electropulsing-assisted rolling process is simple, energy saving, and environmentally friendly, which can effectively improve the processing quality. After electropulsing-assisted rolling, the elongation of materials increases, cracking decreases, deformation resistance reduces, the grain is elongated, texture strength decreases, and microstructure and mechanical properties change significantly.

### 3.4. Electropulsing-Assisted Ultrasound Rolling

**Figure 23:**
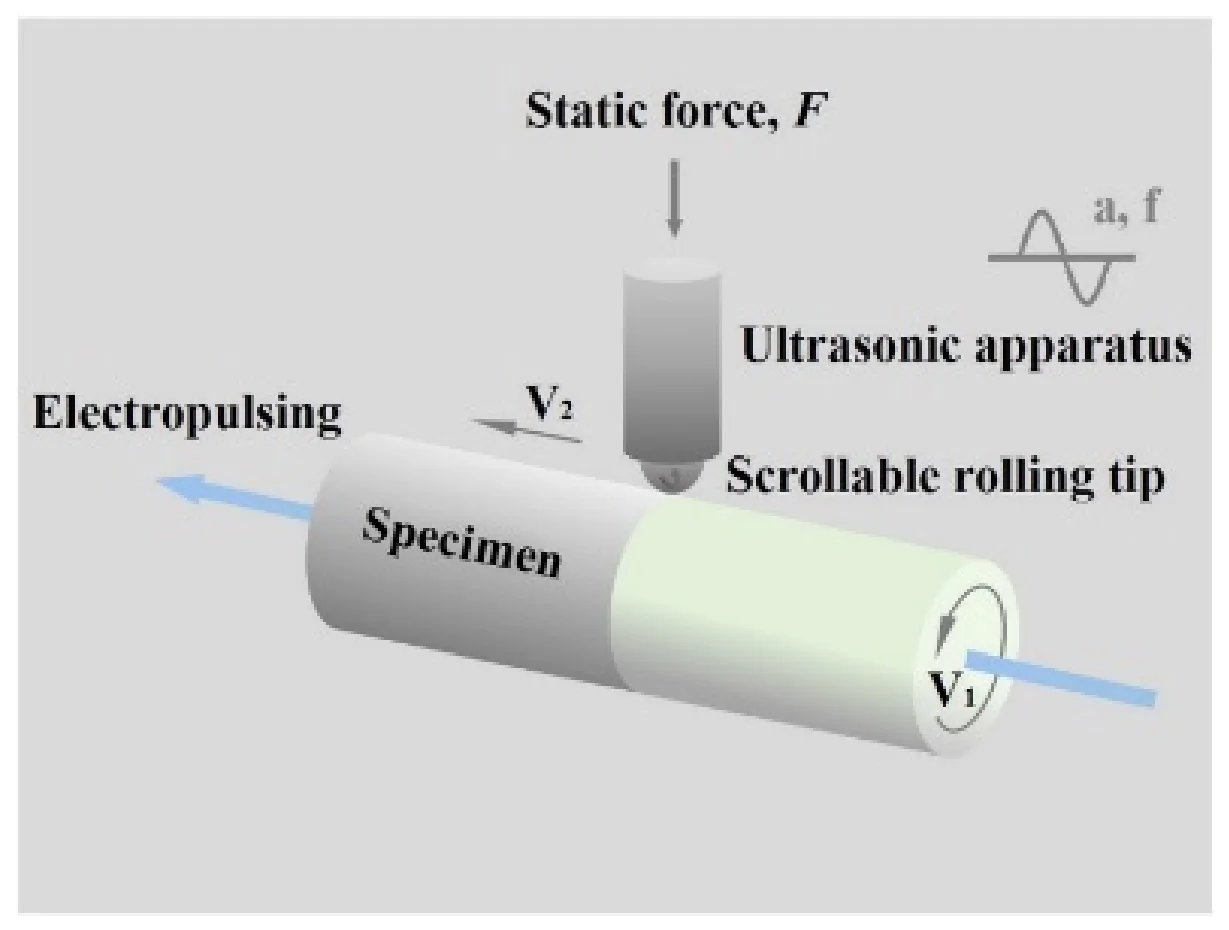
Schematic of the electropulsing-assisted ultrasonic rolling process [175].

Ultrasonic rolling technology is an emerging surface processing technology in recent years. It integrates ultrasonic impact technology into rolling process, injecting intense vibration impact energy into the surface layer of workpiece. This causes the material surface to undergo severe plastic deformation [162,163,164], resulting in work-hardening [165], promoting grain refinement [166,167], generating gradient nanocrystals [168,169], and altering the stress distribution state [170], thereby enhancing the surface and mechanical properties of materials [171,172,173,174]. However, if the ultrasonic amplitude and static pressure are continuously increased, it will cause damage, such as peeling and flaking on the material surface, thereby deteriorating the surface properties and impeding the further enhancement of material properties. Introducing electropulsing into the ultrasonic rolling process leverages the effects of electropulsing to promote atomic diffusion, drive dislocation movement, facilitate grain boundary migration, and enhance the plastic deformation capability of materials, helping to overcome processing limitations and further improving the surface and mechanical properties of materials [24]. The schematic diagram of the electropulsing-assisted ultrasonic rolling process is shown in Figure 23. As can be seen from the figure, electropulsing is applied in the axial direction of the workpiece and static pressure and ultrasonic waves are applied in the radial direction of the workpiece through the rolling head, which is in contact with workpiece. During the working process, the workpiece rotates at a uniform speed and the rolling head moves along the axial direction of the workpiece at a uniform speed.

**Figure 24:**
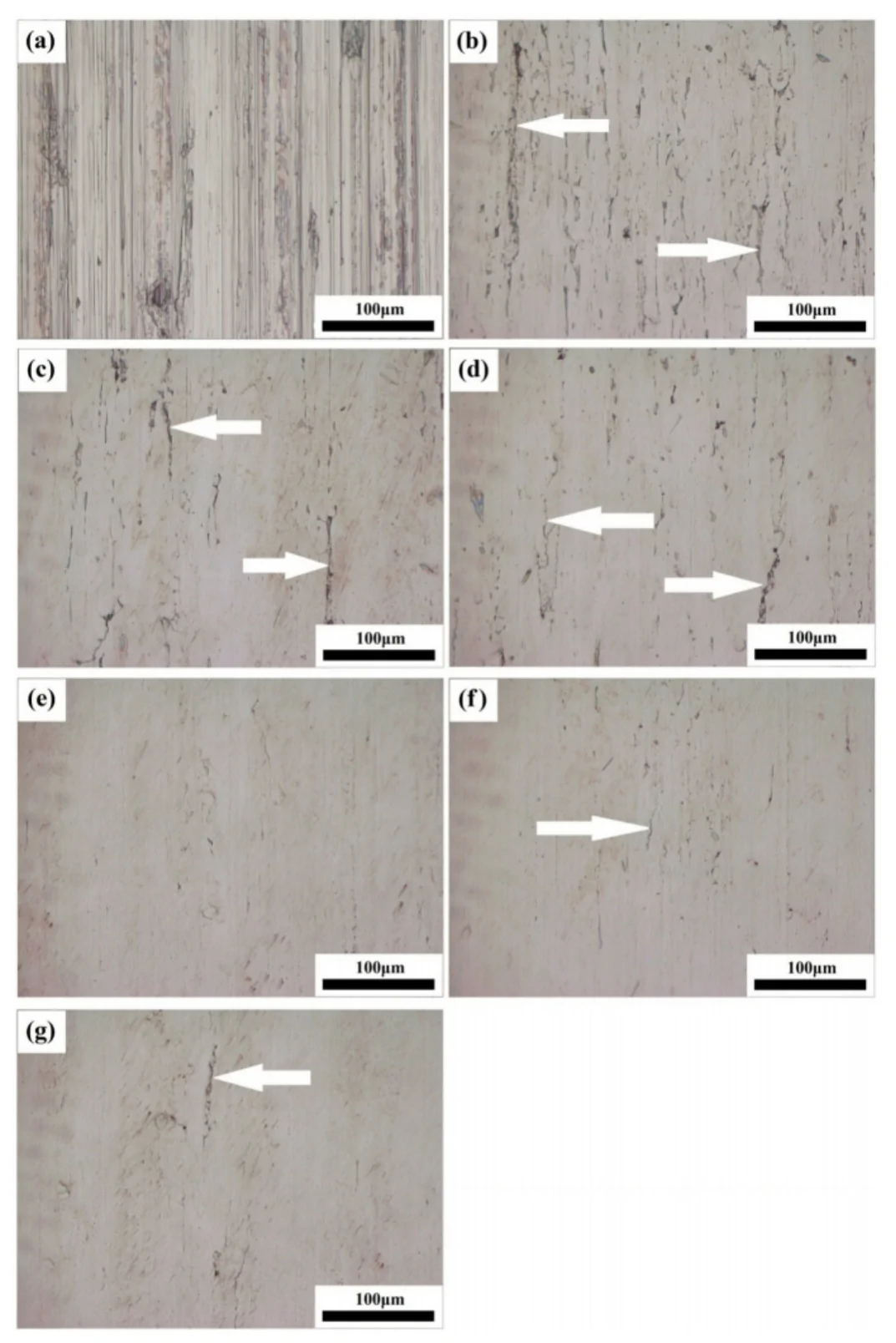
Surface morphology of 304 stainless steel after different processes [63]: (**a**) Turning; (**b**) ultrasonic tumbling; (**c**) 500 Hz; (**d**) 550 Hz; (**e**) 600 Hz; (**f**) 650 Hz; and (**g**) 700 Hz electropulsing-assisted ultrasonic rolling [175].

Electropulsing-assisted ultrasonic rolling helps to increase plastic deformation capacity, reduce surface roughness, increase surface hardness, and improve the surface properties of materials. Gao et al. [44] investigated the effects of electropulsing-assisted ultrasonic rolling on the surface properties of 9310 carburized steel, it was found that the surface roughness of the material in its original state was Ra 3.177 μm. Additionally, it was found that after ultrasonic rolling the surface roughness of the material was reduced by 78% to reach Ra 0.707 μm, while after 50 V electropulsing-assisted ultrasonic rolling, the surface roughness of the material was further reduced, to only 5% of its original value to reach Ra0.154 μm. Meanwhile, the surface microhardness and the depth of the reinforced layer of the material increased significantly after 50 V electropulsing-assisted ultrasonic rolling, the surface microhardness increased about 30% to 909 HV, and the depth of the reinforced layer increased about 56% to 1400 μm. Wang [175] investigated the effect of electropulsing-assisted ultrasonic rolling on the surface properties of 304 stainless steel, they discovered that electropulsing-assisted ultrasonic rolling significantly reduced the surface roughness of material, so that the surface roughness of material was significantly reduced from Ra 1.1457 μm to Ra 0.0934 μm. The surface morphology of material after different machining processes was subsequently observed, as shown in Figure 24. It can be seen from the figure that the surface of the material is relatively rough after turning processing and that the surface quality of the material is clearly improved after ultrasonic rolling process, though there are still many microcracks and defects (pointed by the white arrow); however, when electropulsing is applied, it promotes the healing of microcracks and reduces the number of defects, which further improves the surface quality. when the pulse frequency is 600 Hz, the surface quality of the material is higher and no micro-cracks and defects are observed. The electropulsing-assisted ultrasonic rolling also had a significant effect on surface hardness and depth of reinforced layer. The surface microhardness of the material after ultrasonic rolling was 375 HV and the depth of reinforced layer was about 800 μm; after electropulsing-assisted ultrasonic rolling (600 Hz), the surface microhardness of the material increased to 447 HV and the depth of the reinforced layer increased to 1200 μm.

**Figure 25:**
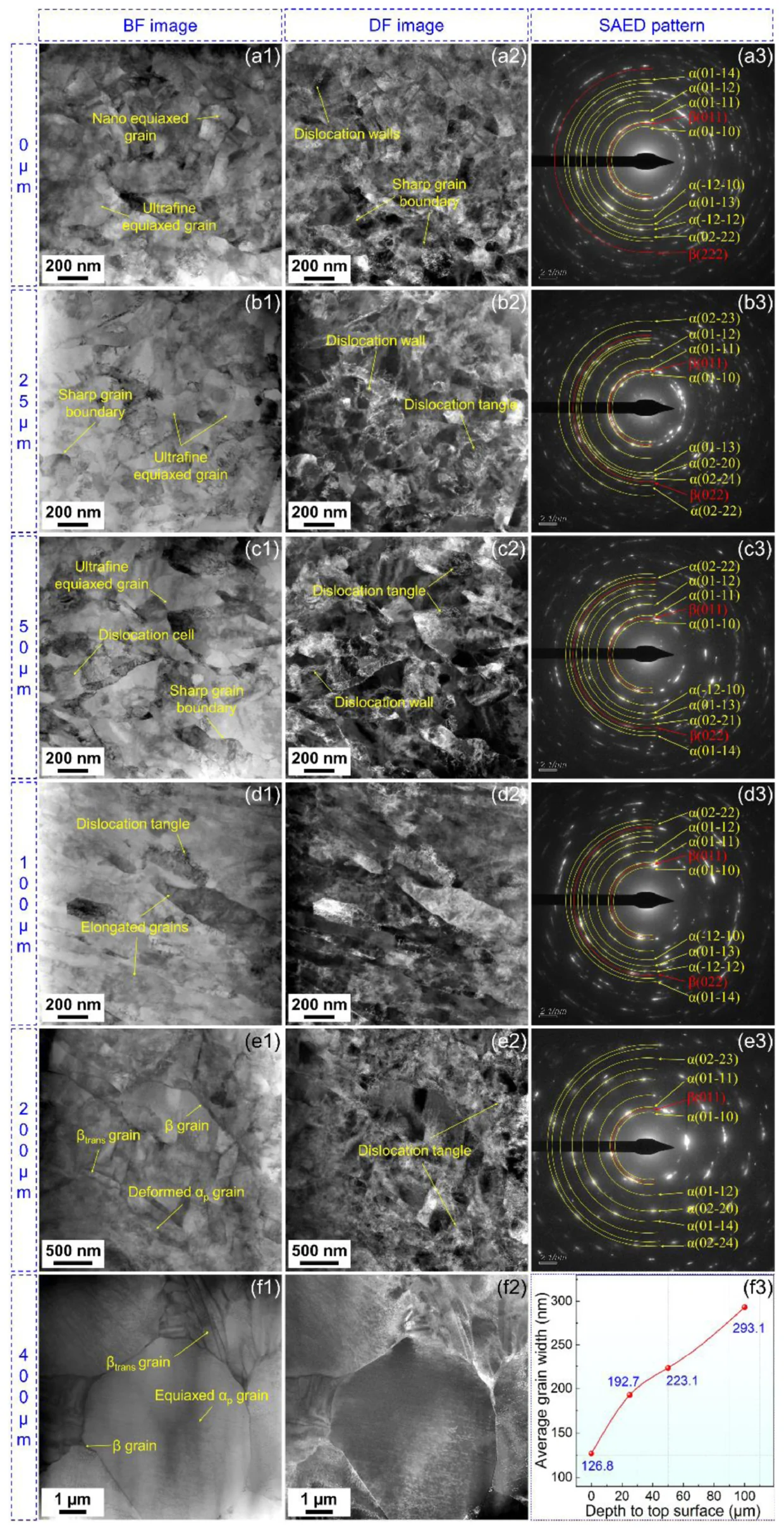
Microstructure of Ti-6Al-4V alloy at different depths from surface after electropulsing-assisted ultrasonic rolling process: (**a1**–**a3**) 0 μm; (**b1**–**b3**) 25 μm; (**c1**–**c3**) 50 μm; (**d1**–**d3**) 100 μm; (**e1**–**e3**) 200 μm; (**f1**,**f2**) 400 μm; (**f3**) grain size variation [45]. Scale bar: 2 1/nm.

Electropulsing-assisted ultrasonic rolling can refine the surface grains and produce gradient nanocrystals, which have a significant effect on the surface microstructure of materials. Sun et al. [45] observed the microstructure of Ti-6Al-4V alloy at different depths from the surface after electropulsing-assisted ultrasonic rolling, as shown in Figure 25. It can be seen from the figure that there are more types of grains at the surface of the material, such as nano-isometric grains, ultrafine isometric grains, ultrafine elongated grains, and widened elongated grains, the dislocation density at the surface is lower; at 25 μm from the surface, there are mainly ultrafine equiaxed grains and ultrafine elongated grains, where dislocation entanglement is more serious, the dislocation density is higher and the grain size of the surface is slightly increased; at 50 μm from the surface, there are mainly ultrafine elongated grains with small grain sizes and clear grain boundaries, indicating that dislocation recovery has occurred here, but dislocation entanglement and dislocation walls indicate that a large number of dislocations still exist; at 100 μm from the surface, there are mainly ultrafine equiaxed grains, ultrafine elongated grains, and a small number of nano-equiaxed grains. The appearance of nano-equiaxed grains indicates that the plasticity of the material is weakened as the ultrasonic rolling process makes the grains fracture; at 200 μm from the surface, there are mainly ultrafine elongated grains and ultrafine equiaxed grains, and no nano-equiaxed grains are observed, indicating that the effect of electropulsing-assisted ultrasonic rolling on grain refinement is weakened here; at 400 μm from the surface, the grain size is larger and the grains show slight deformation, the grain boundaries are clear, while a large number of dislocation entanglements are observed at grain boundaries, indicating that electropulsing-assisted ultrasonic rolling has less of an effect here.

Electropulsing-assisted ultrasonic rolling can change the internal stress distribution state of materials, thus improving their mechanical properties [176]. Wang et al. [177] investigated the variation of residual stresses in AISI 304 stainless steel after different processes, as shown in Table 4. It can be seen from the figure that both the ultrasonic rolling process (USRP) and electropulsing-assisted ultrasonic rolling (EP-USRP) can introduce residual compressive stress within a certain depth of specimen. The compressive residual stress exhibits an increasing trend followed by a decreasing trend along the depth direction and eventually transforms into tensile residual stress. After applying pulsed electric current, the material exhibits a significantly higher level of compressive residual stress compared with the ultrasonic rolling. With increasing pulse current frequency, both the magnitude and depth of the compressive residual stress layer initially increase and then decrease. When the pulse current frequency reaches 600 Hz, the maximum compressive residual stress magnitude and depth are achieved. The maximum value of residual compressive stress in specimen after ultrasonic rolling is about −940 MPa and the acting depth is about 400 μm, while electropulsing-assisted ultrasonic rolling can increase the residual compressive stress and the acting depth in material, the residual compressive stress reaches the maximum value of about −1410 MPa and the acting depth is about 800 μm at the frequency of 600 Hz of the electropulsing current. The higher residual compressive stresses resulted in a significant increase in the fatigue strength of the material. The average rotational bending fatigue strength of the material after ultrasonic rolling was 561.5 MPa, whereas the average rotational bending fatigue strength of the material after electropulsing-assisted ultrasonic rolling was increased to 593.5 MPa.

**Figure 26:**
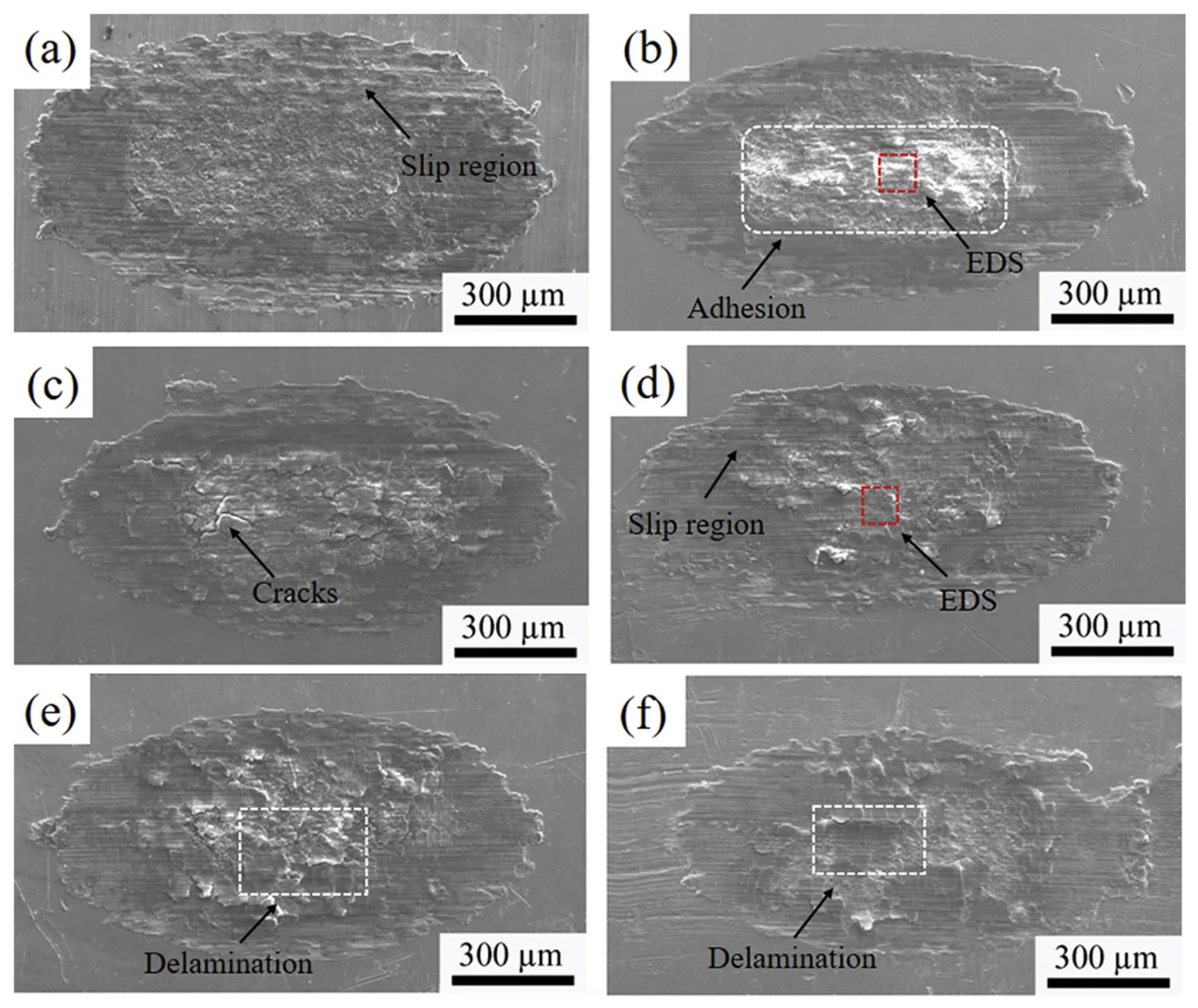
Morphology of wear marks on pure titanium after different processes [63]: (**a**) Turning; (**b**) ultrasonic tumbling; (**c**) 450 Hz; (**d**) 500 Hz; (**e**) 550 Hz; and (**f**) 600 Hz electropulsing-assisted ultrasonic rolling.

Electropulsing-assisted ultrasonic rolling can improve the surface properties and microstructure of materials, improving the friction and wear properties of the materials. Gao et al. [44] investigated the friction and wear properties of 9310 carburised steel after electropulsing-assisted ultrasonic rolling, they discovered that the maximum wear of the material in the initial state was 660.8 μm^2^; after ultrasonic rolling, the maximum wear of the material was reduced about 78.3% to 143.2 μm^2^; after electropulsing-assisted ultrasonic rolling (50 V) the maximum wear of material was reduced about 88.2% to 78.0 μm^2^. Upon subsequent study of the friction coefficient of the surface, it was found that, after 50 cycles, the average friction coefficient of initial material was 3.05; after ultrasonic rolling the friction coefficient of the material was reduced about 58.4% to 1.27; and after electropulsulsing-assisted ultrasonic rolling (20 V), the friction coefficient of the material was reduced about 76.4% to 0.72. Ye et al. [178] observed the abrasion morphology of pure titanium after different processes, as shown in Figure 26. As can be seen from the figure, the surface abrasion area of the turning samples is larger, which is related to the lower surface hardness and the higher surface roughness of the material. After ultrasonic rolling, the area of abrasion marks was reduced and the bonding phenomenon was observed on the surface of sample. This is mainly because ultrasonic rolling improves the surface hardness of the material, forming a reinforced layer on the surface; however, the thickness of the reinforced layer is thin and is easily consumed in the process of friction and wear. After electropulsing-assisted ultrasonic rolling, the surface hardness of the material and the depth of the reinforced layer increased. Additionally, the surface bonding phenomenon was alleviated. With the increase of pulse frequency, the surface abrasion marks are smooth and then rough, which is related to the changes in surface hardness and coefficient of friction.

Electropulsing-assisted ultrasonic rolling changes the microstructure and internal stress distribution state of materials and can also improve the corrosion resistance of materials. Sun et al. [179] discovered that electropulsing-assisted ultrasonic rolling can significantly improve the anti-cavitation corrosion performance of nickel aluminum bronze. This is mainly because electropulsing-assisted ultrasonic rolling improves the surface quality of the material and produces compressive stress on its surface, which effectively resists the impact of cavitation bubbles. Meanwhile, electropulsing-assisted ultrasonic rolling makes the grain in the surface layer become deflected, which leads to the change of crack extension direction, which also effectively improves the corrosion resistance of the material.

In addition, the surface and mechanical properties of several common difficult-to-machine metal materials after electropulsing-assisted ultrasonic rolling are shown in Table 5. In summary, electropulsing-assisted ultrasonic rolling technology is highly efficient and can improve the plastic deformation ability of materials and break through the processing limit of ultrasonic rolling technology. Electropulsing-assisted ultrasonic rolling technology can reduce the surface roughness of the materials, refine surface grain size, form gradient nanostructures, enhance surface hardness, and increase the depth of the deformation layer, so that higher surface quality and excellent mechanical properties of materials are given.

## 4. Conclusions

Difficult-to-machine metal materials are characterized with high strength, high hardness, and good corrosion resistance, and have wide application in the aerospace and defense industries, as well as other fields. Conventional machining technology in the processing of difficult-to-machine metal materials often faces the problem of severe tool wear and poor surface quality, while electropulsing-assisted machining technology can effectively enhance the plastic deformation of difficult-to-machine metal materials, reduce tool wear in the machining process, and obtain high-quality machined surfaces. In recent years, the electropulsing-assisted processing of difficult-to-machine metal materials technology has made significant progress. The prospects for subsequent research in this area are as follows:

(1) Deeper research on the mechanism of the effect of electropulsing on materials. At present, the mechanism of the effect of electropulsing on materials is still unclear, so the next step should focus on the coupling of the thermal and athermal effects of electropulsing and a clarification of the specific contributions of both in promoting atomic diffusion, dislocation evolution, and grain boundary migration. At the same time, attention should be paid to the relative strengths of thermal and athermal effects under different pulse current parameters and their effects on the microstructure and properties of materials.
(2) Optimization process parameters of electropulsing-assisted machining difficult-to-machine metal materials. In recent years, much research has been carried out on electropulsing-assisted machining technology for difficult-to-machine metal materials, but there is still potential for the machining quality of difficult-to-machine metal materials. Next, the parameters of electropulsing should be optimized for different difficult-to-machine metal materials, so as to further improve the machining quality.
(3) The development of new processing technologies for the coupling of electropulsing with other fields. Difficult-to-machine metal materials have broad application prospects and the realization of the high-quality machining of difficult-to-machine metal materials can help to promote this wide application. Therefore, there should be a focus on the development of new machining technologies that couple electropulsing and other fields. This should be undertaken while studying the mechanism of electropulsing and optimizing the process parameters.

## Figures and Tables

**Table 1:** Mechanical properties of 304 austenitic stainless steel after electropulsing-assisted drawing.

Diameter (mm)	Traditional Drawing			Electropulsing-Assisted Drawing		
	σ_b_ (MPa)	σ_0.2_ (MPa)	δ (%)	σ_b_ (MPa)	σ_0.2_ (MPa)	δ (%)
1.50	885	78,201	25	840	690	31
1.40	1010	916	4.0	940	832	15
1.30	1260	1130	2.5	995	889	9
1.20	1420	1282	2.0	1040	937	7
1.13	1490	1326	2.0	—	—	—
1.01	1520	—	—	1100	1095	5
0.91	—	—	—	1150	—	4
0.81	—	—	—	1200	—	3
0.70	—	—	—	1260	—	2
0.62	—	—	—	1360	—	1
0.54	—	—	—	1380	—	1
0.47	—	—	—	1440	—	—
0.40	—	—	—	1640	—	—

**Table 2:** Variation of rolling force during electropulsing-assisted rolling and conventional hot rolling of AZ31 magnesium alloy [147].

Rolling Temperature (K)	Reduction Rate of Rolling Force (Δσ/σ_0_ × 100%)	
	Conventional Hot Rolling	Electropulsing-Assisted Rolling
373	—	1.0
420	1.0	3.7
453	—	5.9
470	3.7	7.4
523	7.4	15.0

**Table 3:** Mechanical properties of several difficult-to-machine metal materials after electropulsing-assisted rolling.

Materials	Frequency (Hz)	Pulse Current Intensity (A/mm^2^)	Temperature (K)	Rolling Passes	Thickness Reduction of each Rolling (%)	Tensile Strength (MPa)	Elongation (%)	Ref.
AZ31	200	350	420	—	25	380	4.5	[147]
	400		470			362	6.5	
	200	270	420			351	4.0	
	400		470			340	5.6	
	200	120	423	—	8	299	6.2	[153]
	400	111	498			298	8.1	
	500	106	523			295	10.9	
	600	101	538			293	7.5	
	700	98	548			293	7.3	
	100	456	478	—	23	387	4.5	[154]
	300	322	493			295	6.3	
	500	261	513			295	5.5	
	700	206	538			300	6.5	
	1000	167	563			300	5.5	
	500	140	493	3	6	350	20	[155]
				5		285	16	
				7		270	15	
	10~1000	—	418	1	16	278	4.4	[156]
			623	4	14	272	3.7	
			603	9	9	239	3.0	
			593	12	7	245	1.9	
			573	16	6	210	2.8	
	500	140	493	11	8	331	—	[157]
AISI 1010 Low-carbon martensitic steel	600	—	<323	—	10	1517	12.9	[152]
	600	—	<323	—	30	1586	10.2	
NiTi	200	178	663	1	40	—	26.8	[158]
ZrTi	500	—	298	—	1.7	1440	2.4	[159]
VT6 alloys	1000	150	—	—	—	1420	6	[160]
CP titanium	1000	95	—	—	90(total)	1015	—	[161]

**Table 4:** Variation of residual stresses in AISI 304 stainless steel after different process [175].

Distance to Surface (μm)	USRP (MPa)	EP-USRP		
		550 Hz (MPa)	600 Hz (MPa)	650 Hz (MPa)
0	−930	−1110	−1330	−1220
30	−940	−1120	−1370	−1240
60	−910	−1100	−1410	−1260
90	−850	−1010	−1410	−1230
140	−670	−790	−1290	−980
200	−510	−670	−1190	−820
300	−270	−520	−880	−630
500	190	50	−480	−290
700	160	70	−140	0
1000	220	160	130	110
1500	80	40	90	20

**Table 5:** Surface and mechanical properties of difficult-to-machine metal materials after electropulsing-assisted ultrasonic rolling.

Materials	Surface Grain Size (μm)	Reinforcement Layer Depth (μm)	Surface Roughness (μm)	Hardness (HV)	Residual Stress (MPa)	Tensile Strength (MPa)	Fatigue Strength (MPa)	Ref.
Carburized 9310 steel	—	1400	0.154	909	—	—	—	[44]
C45E4 steel	0.03~0.05	700	0.25	460	—	—	—	[43]
Austenitic stainless steel	—	600	0.2	423	—	—	—	[180]
AISI 304 stainless steel	—	—	0.0934	447	−279	741	—	[181]
Welded S50C steel	—	400	—	295	−87	—	—	[182]
Pure titanium	<6	>480	0.026	308	−315	—	—	[178]
25CrNi2MoV steel	0.005	100	0.1173	625	−850	—	865	[46]
Ti6Al4V	0.5~2	>100	0.2	423	−554	—	—	[63]
	0.16	165	0.173	417	−733	—	780	[45]
	<0.35	>200	0.019	407	—	—	—	[92]
Ti5Al4Mo6V2Nb1Fe	0.03	260	0.025	432	−880	1167	650	[176]
Entropy CrMnFeCoNi alloy	—	—	—	802	—	—	—	[61]

## Data Availability

No new data were created or analyzed in this study. Data sharing is not applicable to this article.
